# Abstracts from the 4th International PPRI Conference 2019: Medicines access challenge – The value of pricing and reimbursement policies: Vienna, Austria. 23-24 October 2019

**DOI:** 10.1186/s40545-019-0194-x

**Published:** 2019-10-18

## Editorial

### Can pharmaceutical pricing and reimbursement policies make a difference in promoting equitable access to affordable medicines? From diagnosis to sustainable impact

#### Sabine Vogler^1^, Nina Zimmermann^1^, Manuel Alexander Haasis^1^, Zaheer-Ud-Din Babar^2^, Reinhard Busse^3^, Jaime Espin Balbino^4^, Aukje Mantel-Teeuwisse^5^, Fatima Suleman^6^, Veronika J. Wirtz^7^

##### ^1^WHO Collaborating Centre for Pharmaceutical Pricing and Reimbursement Policies, Pharmacoeconomics Department, Gesundheit Österreich GmbH (GÖG / Austrian Public Health Institute), Vienna, Austria; ^2^University of Huddersfield, Huddersfield, United Kingdom; ^3^Department of Health Care Management, Technische Universität Berlin, Berlin, Germany; ^4^Andalusian School of Public Health (EASP), Granada, Spain; ^5^WHO Collaborating Centre for Pharmaceutical Policy and Regulation, Division of Pharmacoepidemiology & Clinical Pharmacology, Utrecht Institute for Pharmaceutical Sciences (UIPS), Utrecht University, the Netherlands; ^6^WHO Collaborating Centre for Pharmaceutical Policy and Evidence Based Practice, Discipline of Pharmaceutical Sciences, School of Health Sciences, University of KwaZulu-Natal (Westville Campus), Durban, KwaZulu-Natal 4000, South Africa; ^7^WHO Collaborating Centre in Pharmaceutical Policy, Department of Global Health, Boston University School of Public Health, Boston, Massachusetts, USA

The 2015 Pharmaceutical Pricing and Reimbursement Information (PPRI) Conference presented major challenges in achieving equitable access to affordable medicines not just in low- and middle-income countries but in high-income countries as well. This included innovative medicines such as sofosbuvir whose planned market entry hit public payers of high-income countries unprepared. As further new medicines with high prices were expected to come to the market in the future, the “sofosbuvir case” can be seen as a kind of “wake-up” call.

Apart from high-priced medicines that dramatically challenge the sustainability of pharmaceutical systems, discussions at the 2015 PPRI Conference included non-availability of effective low-priced medicines, critical assessment of the intended and unintended effects of existing policies and supportive tools such as cost-effectiveness analysis as well as limitations related to transparency in medicine price information [1].

**Local challenges, global learnings?**

Compared to previous years, the 2015 debate was characterized by the fact that all countries, including rich economies, were struggling to ensure affordable medicine access to their citizens. Promoting affordable medicines used to be an individual fight, since pharmaceutical policies for procuring, pricing and funding medicines are national competence – even in the countries of the European Union that harmonised the regulatory framework for marketing authorisation.

However, driven by a move to “globalising solidarity”, significant changes occurred between the previous and the current 2019 PPRI Conference. Authorities and payers took action to systematically work collaboratively in technical areas, such as horizon scanning, joint negotiations and procurement. In Europe, cross-country collaborations such as the Valetta Declaration or the Beneluxa initiative were established [2, 3]. The European Commission tabled a proposal of how to organise health technology assessment in Europe in a sustainable manner, especially for innovative pharmaceuticals [4]. Informal collaborations increased in recent years, which provide a platform for pricing and reimbursement authorities for an exchange of best practices and experiences with policy implementation, such as the PPRI network [5], allowing for cross-country learnings on best practices. Lessons from these initiatives are presented at the 2019 PPRI Conference.

Since 2015, the pharmaceutical policy world has seen the adoption of some Council Conclusions of the European Union related to the challenge of high-priced medicines [6, 7], the report of The Lancet Commission on Essential Medicines [8], the report of the UN Secretary General’s High-Level Panel on Access to Medicines [9], the establishment of the “Fair Pricing Forum” by the WHO [10] and the adoption of a WHO resolution on transparency [11]. Though there are differences in wording and detail, all these initiatives aimed to develop new models, based on the principles of equity, fairness, accountability and transparency of medicine prices and R&D costs, for ensuring access to innovative medicines. The discussion panels at the PPRI Conference will examine these proposals and identify opportunities for further adaptations of these new models where necessary.

**“Fake” prices – Are price surveys still useful?**

During the last few years, policy-makers and payers have increasingly become frustrated over managed-entry agreements that were initially perceived as a promising policy option, but are instead used as an instrument as the last resort. The “price” that governments pay for making medicines available to their citizens is to agree into confidential arrangements, though signalling a high list price to other countries. As a result, authorities are frequently confronted with published price information that is flawed. This poses new challenges as more countries apply external price referencing (EPR) because several middle-income countries started to regulate medicine prices and EPR is the preferred pricing policy.

While many policy-makers and payers around the globe have become aware of the weakness of existing pharmaceutical policies such as EPR, managed-entry agreements and value-based pricing, recent years have also seen advances in methodologies applied in pricing and reimbursement policies, e.g. use of multi-criteria decision analysis for decision-making [12], guiding principles for a well-chosen methodological design in EPR [13]. As long as no concrete or well-defined solution to the medicines access challenge is implemented, a well-designed mix of existing and further developed policies is the next best option. These policies need to be tailored to the different types of medicines and to the country context. Despite their limitations, adequate pricing and reimbursement policies offer value in promoting equitable access to affordable medicines. Advanced methodologies and new evidence, including work presented at the 2019 PPRI Conference, should be considered.

**Pharmaceutical systems research at the interface of diagnosis and action**

In this respect, pharmaceutical systems research (PSR) can make a valuable contribution. PSR is a new discipline that derives from health systems research. Through descriptive case studies, it addresses topics such as the organisation and funding of pharmaceutical systems, policies (e.g. related to pricing, reimbursement, distribution and rational use of medicines), actors (e.g. authorities, stakeholders) and implementation procedures. Comparative cross-country studies, either descriptive or analytic, help improve the pharmaceutical systems of different settings, in terms of affordability, efficiency and quality [14]. Finally, impact evaluations study the effects of policy implementation; using a pharmaceutical system lens can augment the policy relevance of these evaluations. In fact, PSR is a policy-supporting area of science that can support to work on solutions or, at least, improvements in the pharmaceutical policy framework of individual countries and globally.

**Fixing the future**

Is it sufficient to have debates, policy papers, scientific evaluation, methodology advancement, cross-country best practices learnings and exchange experiences to improve pricing and reimbursement policies in Europe and other regions? Each of these pathways taken by policy-makers, payers, researchers and/or stakeholders offers value. Nonetheless, we need a combination of all to arrive from a thorough diagnosis to sustainable impact: we need wise and transparent policy-making, robust and multi-disciplinary science, critical assessment of existing policies and tools as well as frank and in-depth discussions. The 2019 PPRI Conference makes a significant contribution to providing a platform for these activities that are critical to promote equitable access to affordable medicines.

**References**

1. Vogler S, Zimmermann N, Ferrario A, Wirtz VJ, de Joncheere K, Pedersen HB, et al. Pharmaceutical policies in a crisis? Challenges and solutions identified at the PPRI Conference. Journal of Pharmaceutical Policy and Practice. 2016;9(1):1.

2. Espín J, Rovira J, Calleja A, Azzopardi-Muscat N, Richardson E, Palm W, et al. How can voluntary cross-border collaboration in public procurement improve access to health technologies in Europe? Policy Brief. 2016;21. Available from: https://www.eu2017.mt/Documents/Programmes/PB21.pdf (accessed 1 March 2019).

3. Vogler S, Paris V, Panteli D. Ensuring access to medicines: How to redesign pricing, reimbursement and procurement? Policy Brief 30. Copenhagen: World Health Organization, 2018.

4. European Commission. Proposal for a Reguatlion of the European Parliament and of the Council on health technology assessment and amending Directive 2011/24/EU. Available from: https://ec.europa.eu/health/sites/health/files/technology_assessment/docs/com2018_51final_en.pdf (accessed 6 February 2018). Brussels: 2018.

5. Vogler S, Leopold C, Zimmermann N, Habl C, de Joncheere K. The Pharmaceutical Pricing and Reimbursement Information (PPRI) initiative–experiences from engaging with pharmaceutical policy makers. Health Policy and Technology. 2014;3(2):139–48.

6. Council of the European Union. Council conclusions on strengthening the balance in the pharmaceutical systems in the EU and its Member States. 17 June 2016. Available from: https://www.consilium.europa.eu/en/press/press-releases/2016/06/17/epsco-conclusions-balance-pharmaceutical-system/ (accessed 1 March 2019).

7. Council of the European Union. Council conclusions on Encouraging Member States-driven Voluntary Cooperation between Health System. 16 June 2017. Available from: http://data.consilium.europa.eu/doc/document/ST-10381-2017-INIT/en/pdf (accessed 1 March 2019).

8. Wirtz VJ, Hogerzeil HV, Gray AL, Bigdeli M, de Joncheere K, Ewen MA, Gyansa-Lutterodt M, Jing S, Luiza VL, Mbindyo RM, Möller H, Moucheraud C, Pécoul B, Rägo L, Rashidian A, Ross-Degnan D, Stephens PN, Teerawattananon Y, ‘t Hoen EFM, Wagner AK, Yadav P, Reich MR. Essential medicines for universal health coverage. The Lancet 2017; 389 (10067): 403–476.

9. United Nations Secretary General's High-Level Panel on Access to Medicines. Report of the United Nations Secretary General's High-Level Panel on Access to Medicines. Promoting innovation and access to health technologies. September 2016.

10. WHO. Fair Pricing Forum. 2017 Meeting Report. Geneva: World Health Organization, 2017. Available from: http://www.who.int/medicines/access/fair_pricing/FairPricingForum2017MeetingReport.pdf?ua=1 (accessed 1 March 2019).

11. WHA. Improving the transparency of markets for medicines, vaccines, and other health products. Resolution. Geneva: World Health Assembly, 28 May 2019.

12. Angelis A, Kanavos P. Multiple Criteria Decision Analysis (MCDA) for evaluating new medicines in Health Technology Assessment and beyond: The Advance Value Framework. Social Science & Medicine. 2017 (188): 137-156

13. Vogler S. (editor): Medicine Price Surveys, Analyses and Comparisons. Evidence, Methodology and Guidance. Elsevier,2018

14. WHO Collaborating Centre for Pharmaceutical Pricing and Reimbursement Policies. Pharmaceutical systems research. Vienna: 2019. Available from: https://ppri.goeg.at/pharmaceutical_systems_research (accessed 28 August 2019).

## KEYNOTE SPEAKER PRESENTATIONS

### K1 Global Access to Medicines Challenge. Time for a new approach?

#### Ellen ‘t Hoen (ellenthoen@medicineslawandpolicy.net)

##### Medicines Law & Policy, Amsterdam, The Netherlands

Patents and other forms of exclusive rights, such as data exclusivity and market exclusivity, are meant to stimulate innovation by rewarding inventors with temporary monopolies over their innovations. These monopolies enable them to reap economic rewards if they are successful and thus ensure resources are available for yet more the development of new medicines. However, given that exclusive rights are granted over medical innovations, the consequences of monopoly pricing can be significant if a high price means that no access to the treatment is provided to patients or postponed until lower-priced versions of the product are available.

In the nineties, we have seen the consequences of the system in global health when 8000 people living with HIV/AIDS were dying each day in the developing world while lifesaving medicines were available in wealthier nations but only at very high prices. Even when more affordable generic antiretroviral medicines (ARVs) became available from Indian producers, medicines patents prevented their import and use in many countries. Governments and global institutions found solutions for this problem. The use of flexibilities in patent law [1] and later the availability of patent licenses from the Medicines Patent Pool [2], coupled with the WHO prequalification of ARVs, ensured widespread availability of low priced ARVs. Today, the WHO recommended fixed-dose combination HIV medications are available for less than US$ 70 per patient per year.

Increasingly, high-income countries too, struggle to deal with high medicines prices. Health ministers find it difficult to obtain good results in price negotiations with companies that hold strong monopoly rights. It is therefore not surprising that patients and their physicians call on governments to make use of the same patent law flexibilities that helped access more affordable HIV medicines.

The EU has started a review of the pharmaceutical incentive system including of mechanisms that create or expand market exclusivity such as the Supplementary Protection Certificate, Data exclusivity and the Orphan Medicinal Product legislation that provides 10- year market exclusivity [3]. The objective of the review is to ‘strengthen the balance in the pharmaceutical system in the EU and its Member States’. This process offers the EU and its members the opportunity to amend current regulations and adopt policies to ensure a better balance between incentivizing innovation and ensuring people have access to effective new medicines and treatments. A critical discussion that needs to take place is the question of whether high medicines pricing is the most efficient way of incentivizing innovation. As two Dutch ministers wrote in The Lancet a few years ago: “*The system is broken.… Patent and intellectual property exclusivities are the only cornerstone of the current model. Companies can ask the price they like. This will no longer do. We need to develop alternative business models…*[4]

In pharmaceuticals, the importance of striking the right balance between rewarding innovation and ensuring that medicines are available and affordable is particularly critical. Time has come to experiment with developing alternatives to the reliance on high medicines pricing to finance innovation.

**References**

1. ‘t Hoen EFM, Veraldi J, Toebes B, Hogerzeil HV. Medicine procurement and the use of flexibilities in the Agreement on Trade-Related Aspects of Intellectual Property Rights, 2001–2016. Bulletin of the World Health Organization. 2018 Mar 1;96(3):185. Available: https://www.ncbi.nlm.nih.gov/pmc/articles/PMC5840629/

2. www.medicinespatentpool.org

3. https://www.consilium.europa.eu/en/press/press-releases/2016/06/17/epsco-conclusions-balance-pharmaceutical-system/

4. Ploumen, Lilianne, and Edith Schippers. “Better life through medicine—let’s leave no one behind”. *The Lancet* 389.10067 (2017): 339-341. Available : http://www.thelancet.com/journals/lancet/article/PIIS0140-6736(16)31905-5/fulltext

### K2 Local challenges, global learning: are findings about pricing and reimbursement policies applicable and transferable from one context to another?

#### Veronika J. Wirtz (vwirtz@bu.edu)

##### World Health Organization Collaborating Center in Pharmaceutical Policy, Department of Global Health, Boston University School of Public Health, Boston, USA

The development and implementation of policies and their effects – regardless of pricing, reimbursement, or other policies – is highly context specific, often with large variations between settings (1). Differences in disease prevalence, availability of financial and human resources, legislation, and values represent only some of the factors contributing to these observed variations, making it difficult to analyze the complex pathways leading to change in policy outcomes (2). Given the multiple influencing factors, the question remains as to whether or not findings about pricing and reimbursement policies are applicable and transferable from one context to another. For the purpose of this paper, applicability means whether the intervention process could be implemented in the local setting regardless of outcome and transferability refers to whether it would be as effective in the new setting as it was in the original study setting (3).

Policy research commonly utilizes case studies to allow in-depth, multi-faceted explorations of complex issues in real-life settings through qualitative methods. Another commonly used study design within this field is a cross-national study consisting of individual case studies that are analyzed comparatively (2), allowing for general conclusions about particular pricing or reimbursement policies. Two critical elements to allow testing for applicability and transferability are a (i) detailed description of the process and contextual factors that contributed to the observed effects and (ii) an iterative analysis of cases that are then compared and contrasted to develop general conclusions.

Both steps require the use of a shared taxonomy when describing context. Differences in taxonomy can inhibit common understanding and create barriers to determining applicability and transferability (4). Since pricing and reimbursement policy research is a relatively recent and evolving field of inquiry, there are several important gaps in a standardized terminology. The Pharmaceutical Pricing and Reimbursement Information (PPRI) network has promoted a standardized terminology (5), which is an important milestone in allowing transferability from a local to a regional, European level. To increase global accessibility to this valuable lexicon, it is important to continue the development of a shared taxonomy that is acceptable and applicable across settings, and this will require consensus building among increasingly larger groups of stakeholders.

Finally, in order for this taxonomy to remain relevant in evolving health systems, it is necessary to continuously update the language and drop outdated terms. This type of monitoring might be achieved through a standing technical working group composed of global experts and other invested institutions.

**References**

1. Weyrauch V, Echt L, Suliman S. Knowledge into policy: Going beyond “Context matters”. London: INASP, Politics & Ideas, 2016. Available at: http://www.politicsandideas.org/wp-content/uploads/2016/07/Going-beyond-context-matters-Framework_PI.compressed.pdf

2. Gilson L. 6. Cross-national analysis. In: Health Systems and Policy Research. A Methodology reader. Geneva: Alliance of Health Policy and Systems Research & World Health Organization, 2012.

3. Wang S, Moss JR and Hiller JE. Applicability and transferability of interventions in evidence-based public health. *Health Promotion International 2006;* 21: 76–83.

4. Organization for Economic Cooperation and Development. Proposal for a Taxonomy of Health Insurance. OECD Health Project. Paris: OECD, 2004. Available at: https://www.oecd.org/health/health-systems/31916207.pdf

5. WHO Collaborating Centre for Pharmaceutical Pricing and Reimbursement Policies. Glossary of pharmaceutical terms. Update 2013. Vienna; 2013. Available at: https://ppri.goeg.at/methodology_documents

### K3 Managed-Entry Agreements: standalone novel or part of a series with an open ending?

#### Inneke Van de Vijver (inneke.vandevijver@riziv-inami.fgov.be)

##### National Institute Health & Disability Insurance, Brussels, Belgium

It is not a secret that the upcoming years will be hot ones in the pharmaceutical market. Prices of the newest drug generation rising dramatically has been the rule for some time, and there's no sign of anything changing. Pharmaceutical prices – whether reference is made to list prices or hidden nett prices - no longer reflect the value of the product. The question should be raised if prices ever have reflected the true value of medicines and how long this ‘bubble of inflated prices’ will hold.

All stakeholders are responsible for creating the current delicate situation. It can probably be argued that there was never any response by the payer until the sustainability of the health care system was endangered and the expenditures were in fact already derailing. In order to get a better grip on the ever-increasing pharmaceutical expenditures, several instruments were created over time, more or less independently of each other. Policies such as external price referencing (ERP), managed entry agreements (MEA), maximum prices, price reductions, claw-back agreements and preferential policies are applied worldwide. When new budgetary problems arise, payers are often forced to look for other methods, which only later turn out not to be the ultimate solution either. At the same time, instruments such as end-of-patent policies, ERP and MEA are becoming interlinked and interact, sometimes in a conflicting way, and the advantages of one system appear to be a disadvantage for another. These policies are in fact reactive control mechanisms; maybe a logical consequence of the offer-based systems that are in place. Although on a short-term, the goal of decreasing expenditures is often achieved, no durable solution has yet been found.

An integrated solution is necessary on three different but highly related components: 1) pricing, 2) financing/reimbursement techniques, and 3) budgeting. Whereas focus nowadays mostly lies on improving reimbursement and financing of pharmaceuticals due to high prices (reactive), focus must shift towards budgeting and demand-driven policy development based on horizon scan outcomes (proactive). Therefore, a partnership of all stakeholders, including health care providers (HCPs), industry, patients and payers is needed. Discussions should be transparent and constructive. HCPs and patients should be willing to talk about budget impact, budget limitations and making choices. Industry should engage in discussions on pricing and feasible budgets. And payers should be in the driver’s position with a clear view on where they want to go and what they are willing to pay for it.

Only with an integrated, transparent and thoughtful solution list prices will regain their value and credibility.

### K4 The need for better HTA methodology for more complex and personalised medicine

#### Wim G. Goettsch (w.g.goettsch@uu.nl)

##### WHO Collaborating Centre for Pharmaceutical Policy, Division of Pharmacoepidemiology and Clinical Pharmacology, Utrecht University and National Health Care Institute, Diemen, The Netherlands

Much of the function of HTA and the use of its outputs in healthcare systems has advanced mostly organically in the past decades. It has been reactive to political, societal and financial needs rather than being proactively ‘designed’ to address the needs of diverse and changing healthcare systems. That may also explain why the current use of HTA – as well as how its principles are applied – as supporting tools for making decisions on reimbursement/procurement and use of (new) health technologies in many countries still predominantly focus on the clinical, and sometimes health economic evaluation, of single technologies.

At the same time, the treatment of patients has become much more complicated due to the development of tailored innovative health technologies including combinations of technologies, co-dependent technologies and personalised medicine. Although this personalised approach is in essence desirable, a big issue is that these innovative health technologies with skyrocketing prices and only limited information on their effectiveness and cost-effectiveness will come to patients before there is a clue in which patients these treatments actually work the best. A recent example of such innovative health technology is the CAR-T therapy for acute lymphoblastic leukaemia for which data from clinical practice [1] now show that this treatment may not be as curative as being claimed on the basis of the regulatory trials [2].

Therefore there is growing need for HTA that is capable of identifying for whom health technologies work and for whom they are not essential, hereby guaranteeing that the right treatment is provided, to the right patient, at the right time and leading to an increase in societal healthcare benefits [3]. Therefore, if HTA organisations are expected to make more tailored decisions on complex health technologies using more complicated data, new HTA methods need to be developed for this next generation of healthcare.

To support the development of these methods, a new H2020 project called HTx was started this year. HTx will facilitate the development of methodologies to deliver more customized information on the effectiveness and cost-effectiveness of complex and personalised combinations of health technologies. Additionally, these methods should also enable personalised treatment advice that will be shared between patients and their physicians. Finally, the implementation of these methods can only be realised if we carefully test, validate and use the methods in HTA practice. This effort will be accomplished in close collaboration with the European Network for HTA (EUnetHTA) and its stakeholders.

**References**

1. Schulthess D, et al., Are CAR-T therapies living up to their hype? A study using real-world data in two cohorts to determine how well they are actually working in practice compared with bone marrow transplants. BMJ Evid Based Med, 2019.

2. Maude SL, et al., Tisagenlecleucel in Children and Young Adults with B-Cell Lymphoblastic Leukemia. N Engl J Med, 2018. 378(5): p. 439-448.

3. Love-Koh J, et al., The Future of Precision Medicine: Potential Impacts for Health Technology Assessment. Pharmacoeconomics, 2018. 36(12): p. 1439-1451.

## ORAL PRESENTATIONS - STRAND 1

### O1 Differences in health technology assessment recommendations for pharmaceuticals between European jurisdictions: the role of practice variations

#### Rick Vreman^1,2^, Aukje K Mantel-Teeuwisse^1^, Anke M Hövels^1^ , Hubert GM Leufkens^1^, Wim G Goettsch^1,2^

##### ^1^Division of Pharmacoepidemiology and Clinical Pharmacology, Utrecht Institute for Pharmaceutical Sciences, Utrecht University, Utrecht, The Netherlands; ^2^The National Health Care Institute (ZIN), Diemen, The Netherlands

###### **Correspondence:** Rick Vreman (r.a.vreman@uu.nl)

**Background:** Health technology assessment (HTA) plays an important role in reimbursement decision-making in many countries, but recommendations vary widely throughout jurisdictions, even for the same drug. This variation may be due to differences in weighing of evidence or due to differences in values, processes or procedures; together called HTA practices.

**Objectives:** To provide insight into the effects of differences in practices on interpretation of inter-country differences in HTA recommendations for conditionally approved drugs.

**Methodology:** We included HTA recommendations for conditionally approved drugs (N=27) up until June 2017 from England/Wales, France, Germany, Netherlands and Scotland. Recommendations and practice characteristics were extracted from these five jurisdictions and this data was validated. The effect of non-submissions, resubmissions and reassessments, cost-effectiveness assessments and price negotiations on changes in the percentage of negative recommendations and interpretation of inter-country differences in HTA outcomes were analyzed with Fisher exact tests.

Region covered: EURO, international level

Time period: 2006-2017

**Results:** The inclusion of cost-effectiveness assessments led to significant increases in proportion of negative recommendations within England/Wales (from 4% to 50%, p<0.01) and Scotland (from 21% to 71%, p<0.01). The subsequent inclusion of price negotiations led to significant reductions in the proportion of negative recommendations in England/Wales (from 50% to 14%, p<0.01), France (from 31% to 3%, p=0.012), and Germany (from 34% to 0%, p<0.01). Results indicated that the inclusion of non- and resubmissions might impact Scottish negative HTA recommendations (from 7% to 21%), but this effect was not significant. No significant effects were observed in The Netherlands, possibly due to sample size.

**Conclusions and lessons learned:** Variations in HTA practices between international jurisdictions can have a substantial and significant impact on conclusions about recommendations by HTA bodies, as exemplified in this cohort of conditionally approved products. Studies comparing international HTA recommendations should carefully consider possible practice variations between jurisdictions.

**Keywords:** Health technology assessment, conditional marketing authorization, HTA practices, relative effectiveness assessment, international

### O2 Relationship between pricing regulations and medicine prices: A multi-jurisdictional comparative analysis of real price indices for pharmaceuticals, 1981-2017

#### Kiu Tay-Teo

##### World Health Organization, Geneva, Switzerland

###### **Correspondence:** Kiu Tay-Teo (tayki@who.int)

**Background:** Over the past decades, authorities responsible for the pricing of medicines have implemented policy reforms to ensure affordability, but to varying extents of implementation and success. Examining whether the degrees of interventions had contributed, or could contribute to, lower medicine prices, is pertinent.

**Objectives:** To assess the macroeconomic trends of medicine prices and their potential relationships with pricing regulations.

**Methodology:** This study examined the historical changes in normalized pharmaceutical prices relative to the prices of all goods and services (i.e. real price index), from 1981 to 2017, in Australia and the United States of America (USA). These countries had contrasting health systems and approaches for managing medicine prices: Australia had implemented series of pricing reforms for its single-payer national pharmaceutical insurance scheme; the USA has a market-based health care, with multiple private and public insurers, and with minimal government intervention on medicine pricing. A separate analysis for the Euro-area countries was conducted based on the harmonized indices on consumer prices and pharmaceutical prices available from 2000. The observed trends were discussed in view of main pharmaceutical pricing reforms in these jurisdictions, identified through a targeted literature review. All data were extracted from statistical authorities in corresponding jurisdictions (1–3).

**Results:** Australia and the USA had similar overall price trends for medicines in the early 1980s, where price index for pharmaceuticals were higher than other consumer goods (Figure 1a). The trends in these countries began to diverge in the late 1980s, where medicine prices continued to rise faster in the USA, while Australia’s real price index started to stabilize at around 10% above the consumer price index. This could be due to introducing “cost effectiveness” as a listing requirement. The divergence became more prominent when further reforms were implemented in Australia from 2005, which resulted in gradual normalization of the growth rate in line with consumer goods. By 2017, the cumulative growth of pharmaceutical prices in the USA reached 2.4 times the overall inflation rate of other consumer goods (Fig.1a). Pricing regulations on medicines in Euro-area countries had also kept medicine prices from rising faster than general consumer prices (i.e. Index<1, Figure 1b)

**Conclusions and lessons learned:** While market and system factors could affect medicine prices, data suggests that higher degree of pricing regulations might have contributed to lower medicine prices in Australia and some Euro-area countries. Laissez-faire policies in the USA seem to have led to unsustainable growth in medicine prices.

**References**

1. Consumer Price Index, Australia. Catalogue number 6401.0 [online database]. Canberra: Australian Bureau of Statistics; 2018 (http://www.abs.gov.au/ausstats/abs@.nsf/mf/6401.0, accessed 15 November 2018).

2. Consumer Price Index [online database]. Washington (DC): Bureau of Labor Statistics of the U.S. Department of Labor; 2018 (https://www.bls.gov/cpi/, accessed 15 November 2018).

3. Harmonised index of consumer prices and detailed average prices [online database]. Luxembourg: eurostat; 2018 (https://ec.europa.eu/eurostat/web/hicp/data/database?p_p_id=NavTreeportletprod_WAR_NavTreeportletprod_INSTANCE_BO6Fgp25CkI9&p_p_lifecycle=0&p_p_state=normal&p_p_mode=view&p_p_col_id=column-2&p_p_col_count=3, accessed 15 November 2018).

**Keywords:** Pharmaceuticals, real price index

**Fig. 1 (abstract O2).**
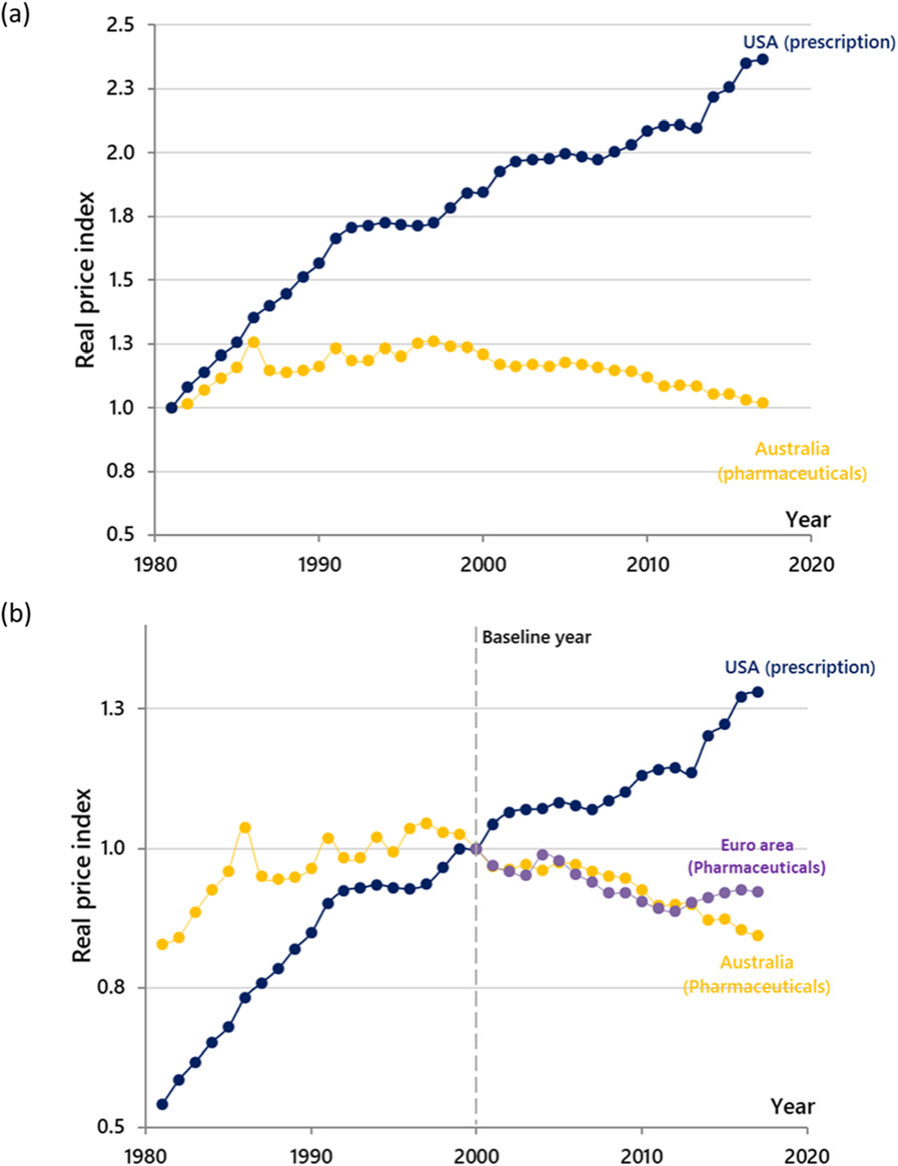
Cumulative real pharmaceutical price inflation in (a) Australia and US with year 1981 as the baseline year (b) Australia, US and Euro area with year 2000 as the baseline year. Note: The index was calculated by dividing the pharmaceutical price index by all-item consumer price index in the baseline year; the term “Euro area” comprises EA11-2000, EA12-2006, EA13-2007, EA15-2008, EA16-2010, EA17-2013, EA18-2014, EA19. Source: Author’s calculation based on data published by the Governments of Australia (1), the USA (2) and Eurostat (3)

### O3 Promoting access to cancer medicines in Mexico: Seguro Popular key policy components

#### Daniela Moye-Holz^1^, Anahi Dreser^2^, Octavio Gómez-Dantés^2^, Veronika J. Wirtz^3^

##### ^1^Department of Community and Occupational Medicine, Medical University Center Groningen, University of Groningen, Groningen, the Netherlands; ²Center for Health Systems Research, Instituto Nacional de Salud Pública (INSP)/National Institute of Public Health, Cuernavaca, Mexico; ³Department of Global Health, University of Boston School of Public Health, Boston, USA

###### **Correspondence:** Daniela Moye-Holz (danymoyeholz@gmail.com)

**Background:** In 2003, Mexico reformed its health system, leading to the creation of Seguro Popular (SP) to reach universal health coverage (UHC). SP provides coverage to basic healthcare and medicines as outlined in its Universal Health Catalogue, and coverage of a package of high-cost interventions through the Fund against Catastrophic Diseases (FPGC), including cancer care and medicines for all children’s cancers and most prevalent adult cancers.

**Objectives:** To describe how SP has addressed the four main components, as described by the World Health Organization (WHO) Access Framework [1], to provide access to cancer medicines: *selection, pricing and reimbursement, financing*, *and procurement and supply systems*.

**Methodology:** This study presents a policy analysis comprising: 1) a document analysis of data, policies, laws, and other relevant information and documentation publicly available in government websites; 2) a pharmacy survey following the WHO/HAI methodology that collected medicines availability and price data [2]; 3) stakeholder interviews in surveyed facilities.

Region covered: Mexico

Time period: 2017

**Results:** 1) The *selection* of cancer medicines is defined by FPGC’s treatment protocols, and SP’s procurement and reimbursement guidelines. SP covers more than 90% of medicines listed in the WHO essential medicines list for cancer. Twenty-eight percent of medicines covered by FPGC are cancer medicines and these provide basic cancer care. 2) SP’s procurement and *reimbursement* guidelines define reference *prices*, which are similar or lower to international reference prices. Health facilities have procured cancer medicines at similar prices as those indicated in the SP guidelines; however, some medicines have been procured at higher prices. 3) SP is *financed* through federal and state government contributions. SP allocates 8% of pooled resources to the FPGC. Cancers represent 10 to 15% of all cases covered by FPGC but represent 30 to 48% of total funds paid. 4) To receive reimbursement from SP, health facilities have to be accredited; there are insufficient facilities accredited to deliver cancer care to satisfy demand. Health facilities use different *procurement and supply mechanisms*: tenders and centralized procurement; outsourced pharmacy services; hybrid model; and direct purchases.

**Conclusions and lessons learned:** SP has addressed the major components outlined by the WHO Access Framework. It is necessary to gradually expand accreditation of facilities and cancer care coverage due to increasing demands. These actions can strengthen the health system and advance UHC, but these should take into consideration the financial resources necessary to maintain the financial sustainability of the system.

**References**

1. World Health Organization. Equitable access to essential medicines: a framework for collective action. WHO Policy Perspect Med. 2004;6.

2. Moye-Holz D. Access to Innovative Medicines in a Middle-Income Country. The Case of Mexico and Cancer Medicines. University of Groningen; 2019.

**Keywords:** access to medicines, cancer, Seguro Popular, Mexico, WHO Access Framework

### O4 Biosimilars in Canada: current environment and future opportunity

#### Tanya Potashnik, Elena Lungu

##### Patented Medicine Prices Review Board, Policy Development, Ottawa, Canada

###### **Correspondence:** Tanya Potashnik (tanya.potashnik@pmprb-cepmb.gc.ca)

**Background:** Potential savings from biosimilars is a subject of keen interest internationally, with a particular emphasis for Canadians in light of the patent life extensions for biologics negotiated under the Canada US Mexico trade agreement. Biosimilars offer an opportunity for significant cost savings, as Canada is a relatively high-use and high-price market for biologics.

**Objectives:** To provide decision makers, researchers, and patients with information on the opportunities in the Canada biosimilars market, as compared with international practices.

**Methodology:** Capturing data from various sources, including the IQVIA MIDAS™ Database, the FDA, EMA, and Health Canada, this presentation compares the emerging Canadian market for biosimilars with our international counterparts. The analysis delves into international comparisons of biosimilar availability, uptake, and pricing, and assesses the potential savings from biosimilars for select recent and upcoming launches.

Region covered: International markets examined include the countries in the Organisation for Economic Co-operation and Development (OECD), highlighting Canada and its comparator markets.

Time period: The analysis focuses on 2018, with retrospective trends back to 2009.

**Results:** Biologics are an important segment of the Canadian pharmaceutical market with annual national sales of $7.7 billion, or nearly one third of pharmaceutical sales in 2018. While the international experience with biosimilars has many success stories – marked by early biosimilar approvals and market entry, and sizable price discounts and uptake – the market dynamics in Canada have been less encouraging. Canadian biosimilar uptake lags well behind Europe, and their prices are often above international norms given the higher prices of the reference biologics prevailing in Canada. For example, in the last quarter of 2018, infliximab biosimilars in Canada were on average 17% more expensive compared to international markets and uptake was only 8.1% (OECD median 36.9%).

With a focus on the challenges in promoting the use of biosimilars in the Canadian market, this research analyses the magnitude of the unrealized savings in Canada and the potential savings for a select number of biologic medicines that could be realized based on the international experience.

**Conclusions and lessons learned:** As the historic savings from generic price reductions and substitutions begin to wane, the potential savings from biosimilars could play in increasing role in offsetting rising drug costs. This overview will uncover the potential for biosimilar savings in Canada, as well as the lessons learned from ongoing Canadian initiatives and the experiences of other jurisdictions.

**Funding Source**

Government of Canada

**Keywords:** Biosimilars, high-cost drugs, Canada

## ORAL PRESENTATIONS - STRAND 2

### O5 Impact of managed-entry agreements on medicines list prices

#### Paolo Pertile^1^, Simona Gamba^2^, Sabine Vogler^3^

##### ^1^Department of Economics, University of Verona, Verona, Italy; ²Department of Economics and Finance, Catholic University, Milan, Italy; ³WHO Collaborating Centre for Pharmaceutical Pricing and Reimbursement Policies, Pharmacoeconomics Department, Gesundheit Österreich GmbH (GÖG / Austrian Public Health Institute), Vienna, Austria

###### **Correspondence:** Paolo Pertile (paolo.pertile@univr.it)

**Background:** Governments have increasingly implemented managed-entry agreements (MEAs) to ensure the market entry of new, high-priced medicines. However, information on their quantitative impact on prices is still extremely scarce. It has been suggested that manufacturers might raise (list) prices in expectation of a MEA to reduce the size of the losses that they could imply [1].

**Objectives:** The study aims to analyse the quantitative impact of the existence of a MEA, and its type (financial- or performance-based), on the list prices of medicines (i.e. before the deduction of any discount).

**Methodology:** A difference-in-difference identification strategy was adopted to estimate the impact of MEAs on ex-factory prices in six European countries (Belgium, England, Italy and the Netherlands = MEA-applying countries; Norway and Greece = no MEA) in December 2016. Publicly accessible information on MEA was retrieved from public authorities; list price data were obtained from the Pharma Price Information (PPI) service. 111 medicines (666 observations) subject to a MEA in at least one of the countries were included in the analysis, and for each medicine, a single pharmaceutical presentation (i.e. a specific pharmaceutical form, dosage and pack size) was selected based on clinical relevance and price data availability across countries.

Region covered: WHO European region

Time period: December 2016

**Results:** Preliminary results show that, on average, the implementation of a MEA increases the list price by 5.2% (significant at the 5% level). The increase is mainly driven by financial-based agreements, which also account for the majority of MEAs in force in December 2016 in the countries of our sample. Controlling for possible heterogeneous effects of MEAs across countries, prices of medicines subject to a MEA in Belgium are 9.5% higher, whereas in Italy and England the effect of the presence of a MEA is statistically lower (Italy: 5.2%, p-value = 0.009; England: 6.1%, p-value = 0.000). The Netherlands is the sole studied country where the effect of a MEA is negative (-7.6%, p−value = 0.038).

**Conclusions and lessons learned:** Preliminary results tend to confirm the hypothesis that the implementation of a MEA increases list prices of medicines. Since we attribute a MEA to a product even when the agreement applies only to a limited number of indications, our estimate of the impact of MEA may be downward biased. The findings imply that payers may overestimate the financial benefits they can obtain from a MEA if they only consider the difference between the list price and the negotiated price net of any discounts.

**Funding Source**

Program ‘Bando di Ateneo per la Ricerca di Base’ funded by the University of Verona

**Reference**

[1] Gerkens S, Neyt M, San ML, Vinck I, Thiry N, Cleemput I. How to improve the Belgian process for managed entry agreements? An analysis of the Belgian and international experience. Brussels: Belgian Health Care Knowledge Centre (KCE), 2017

**Keywords:** managed-entry agreement, medicine list price, difference-in-differences estimation

### O6 European price comparison for patented drugs

#### Melanie Schröder, Carsten Telschow, Jonas Lohmüller

##### WIdO AOK Research Institute, Berlin, Germany

###### **Correspondence:** Melanie Schröder (melanie.schroeder@wido.bv.aok.de)

**Objectives:** This study examines price differences of patented drugs between Germany and comparable European countries. The question is not only how large the average price difference is compared to the individual countries, but also what savings potentials arise for the statutory health insurance (SHI) as a result of these price differences. How does the situation change if statutory manufacturer discounts and collective price negotiations, two political instruments for cost containment in Germany, are taken into account?

**Background:** With over € 38 billion in 2016, pharmaceuticals were the third-largest spending area of SHI, which covers around 90% of Germany's population. Expenses for patented drugs, whose prices have been rising dramatically for years, represent a dominant market share.

**Methodology:** We compare German public list prices for the 250 top-selling patented drugs with list prices in eight European countries collected by systematic online search. To ensure comparability, we take differences in purchasing power into account by adjusting prices. In each case, we calculate savings potentials on a drug-related basis in comparison with the average prices in the countries and the lowest comparative prices.

Region covered: We compared prices in Germany with those in eight other European countries – Belgium, Denmark, Finland, France, Great Britain, the Netherlands, Austria and Sweden.

Time period: Prices were collected as of 1 May 2017, top-selling status of pharmaceuticals refers to revenues in 2016.

**Results:** The adjusted list prices in the reference countries are on average between 18% and 35% below the German list prices. This results in a savings potential of € 3.1 billion, measured by the average ex-factory price in the eight countries, or of € 4.9 billion, measured by the lowest comparative price. This corresponds with a theoretical savings potential of 26.1% for the market segment considered. Taking into account statutory discounts and collective rebates, a conservative estimate still suggests a theoretical savings potential of € 1.5 billion for patented drugs in Germany.

**Conclusions and lessons learned:** Our study shows that cost containment measures applied in the German SHI – a pack-related percentage discount and collective price negotiations introduced for new drugs in 2011 – amount to about 50% of the savings potential in a comparison with list prices in European countries. Collective price negotiations in Germany thus ensure price transparency and reduce the differences to other countries. However, this only applies if one does not assume lower prices than the publicly available list prices for the comparison countries.

**Keywords:** price comparison, patented drugs, AMNOG

### O7 Public and philanthropic financial contributions to the development of new active substances: a bibliographic analysis

#### Louise Schmidt, Claudia Wild

##### Ludwig Boltzmann Institute of Health Technology Assessment, Vienna, Austria

###### **Correspondence:** Louise Schmidt (louise.schmidt@hta.lbg.ac.at)

**Background:** Over recent years there have been several attempts to ascertain the extent to which public funding contributes to the financing of the development of pharmaceuticals and there has been a lively discussion regarding the public return on investment.

**Objectives:** To develop a bibliographic methodology for ascertaining the public and philanthropic financial contribution to the development of new phamaceuticals and to test the methodology using a series of case studies.

**Methodology:** Using drug synonyms, specific sources were searched including Orphanet, clinical trials databases, patent databases, PubMed and agency submissions (FDA, EMA, US Securities and Exchange Commission). Academic papers, grey literature or online information were screened for information on public or philanthropic funding relating to projects that took place before the date of marketing authorisation. The websites of funding organisations or charities were subsequently checked for further information. All therapeutic products are developed on the back of considerable basic research into a disease and its genetic basis. To avoid falsely ascribing the costs of basic research into a disease to specific products, we attempted to identify the time point at which a potential treatment solution was identified and included projects/research from this date onwards. Not included were state financial contributions in terms of tax concessions on R&D activities carried out by the pharmaceutical company.

Region covered: EMA approval of new active substances in 2017 was the starting point for identifying case studies. All public and philanthropic financial contributions were included, regardless of the country of funding.

Time period: The study was conducted in 2019.

**Results:** We developed a structured bibliographic methodology for identifying public and philanthropic financial contributions to the development of new pharmaceuticals. The first case study using the developed methodology was of nusinersen, marketed as Spinraza® (a product for treating children and adults with spinal muscular atrophy). Our results show around EUR 165 million of public or philanthropic monies contributed to research into therapies for SMA (i.e. excluding basic research), of which just over EUR 20 million (conservative estimate) is directly attributable to Spinraza®.

**Conclusions and lessons learned:** The public and philanthropic financial contribution to R&D activities is considerable. The issue of a public return on investment should be used as a factor in negotiations regarding price setting.

**References**

Cleary E. et al, Contribution of NIH funding to new drug approvals 2010-2016. www.pnas.org/cgi/doi/10.1073/pnas.1715368115

Stevens A. et al, The role of public-sector research in the discovery of drugs and vaccines. NEJM 2011; 364(6): 563-541.

**Keywords:** Public return on investment, funding, R&D, bibliographic analysis

### O8 Influence of the difference in discounted price between biosimilar and originator product on competition in Sweden’s infliximab and etanercept market

#### Evelien Moorkens^1^, Steven Simoens^1,^ Per Troein^2^, Paul Declerck^1^, Arnold G. Vulto^1^, Isabelle Huys^1^

##### ^1^Department of Pharmaceutical and Pharmacological Sciences, KU Leuven, Leuven, Belgium; ²IQVIA, Solna, Sweden; ³Hospital Pharmacy, Erasmus University Medical Center, Rotterdam, The Netherlands

###### **Correspondence:** Evelien Moorkens (evelien.moorkens@kuleuven.be)

**Objectives:** Economic theory suggests that a lower price will lead to increased uptake of a product. However, previous research indicates that there is only a weak relationship between the biosimilar market share and the difference in list price between the biosimilar and the originator product [1, 2]. Therefore, this study gathered data on differences in discounted prices between biosimilar and originator infliximab and etanercept in the 21 regions of Sweden and examined its influence on biosimilar uptake in these regions.

**Methodology:** For each region, IQVIA™ provided defined daily doses (DDDs) for infliximab and etanercept products and discounted expenditure for infliximab (Q2 of 2012 to Q4 of 2017). For etanercept, rebated national prices per vial were calculated for 1 October 2017 based on list prices from the Dental and Pharmaceutical Benefits Agency [3], and an indicative rebate level of 65% from a conference presentation [4]. A simple regression analysis of biosimilar infliximab market shares on relative differences in discounted price per DDD was conducted for the year 2017. Actual costs of different etanercept products for the regions and government were calculated for the existing gainsharing agreement (regions: 60%, government: 40%).

**Results:** When visually analyzed, a positive non-linear relationship can be seen between the biosimilar infliximab market share in a region and the relative difference in discounted price per DDD of the biosimilar versus the originator product (Figure 1). This relationship then reaches a maximum: biosimilar market shares exceed 76% from a threshold of a 40% difference in discounted price or more. Although non-linear relationships may apply, a simple regression analysis showed that 59% of the variability in biosimilar infliximab market shares can be explained by the difference in discounted price. The estimated actual costs for the regions to use the different etanercept products show only a 4.6% difference between the originator product and the biosimilar. This might explain why some regions are hesitating to switch and others do not reach biosimilar market shares as high as for infliximab.

**Conclusions and lessons learned:** This study showed the influence of differences in discounted price between biosimilar and originator product on biosimilar uptake, with higher biosimilar market shares with increasing differences in discounted price.

**References**

1. Rémuzat C, Dorey J, Cristeau O, Ionescu D, Radiere G, Toumi M. Key drivers for market penetration of biosimilars in Europe. J Mark Access Health Policy. 2017;5(1):1272308.10.1080/20016689.2016.1272308

2. The Impact of Biosimilar Competition in Europe. London: IQVIA; 2018.

3. Sök i databasen - Tandvårds-Läkemedelförmånsverket [Search the database - The Dental and Pharmaceutical Benefits Agency]: Tandvårds- och läkemedelsförmånsverket; 2018 [09/2018] Available from: https://www.tlv.se/beslut/sok-i-databasen.html?tab=3.

4. Befrits G. Leveraging biosimilars for affordable cancer care - what can we learn from the TNF experience? 23rd Congress of the EAHP; Gothenburg: European Association of Hospital Pharmacists; 2018.

**Fig. 1 (abstract O8).**
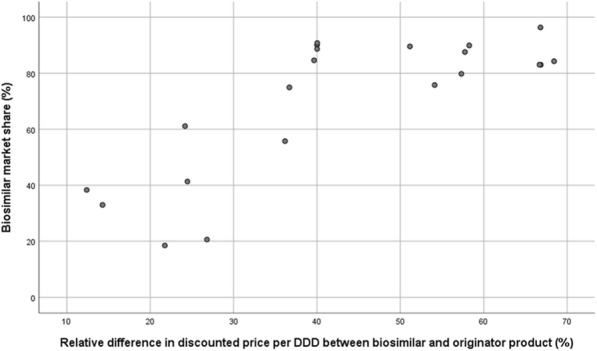
Scatterplot of biosimilar infliximab market shares in the 21 Swedish regions in 2017 and the relative difference in discounted price per defined daily dose (DDD) of the biosimilar relative to the originator product.

### O9 Impact of the external price referencing methodology (EPR) on medicine prices – Simulation of existing EPR models

#### Sabine Vogler, Peter Schneider

##### WHO Collaborating Centre for Pharmaceutical Pricing and Reimbursement Policies, Pharmacoeconomics Department, Gesundheit Österreich GmbH (Austrian Public Health Institute), Vienna, Austria

###### **Correspondence:** Sabine Vogler (sabine.vogler@goeg.at)

**Background:** External price referencing (EPR) is a pricing policy used in several countries to set the prices of medicines. The design of the EPR systems differs between the countries [1]. It can be hypothesised that the methodology of the EPR policy may influence medicine prices in EPR-applying countries.

**Objectives:** The study aimed to investigate the impact of changes in the EPR methodology on medicine prices.

**Methodology:** Discrete-event simulation of fictitious prices based on assumed changes in the EPR methodology were run for a period of 10 years in 28 EPR-applying countries in Europe. The EPR methodology as in place in Q1/2015, whose details were sourced from a survey with competent authorities, was considered as the “base case”. Several dimensions of the EPR policy (consideration of discounts, regular price reviews, adjusting prices by the countries’ income, changes in the basket of reference countries, and changes in the calculation of the reference price and in the exchange rate) were simulated.

Region covered: 28 EPR-applying countries of the WHO European Region (all European Union Member States except Denmark, Sweden, and UK, and Island, Norway and Switzerland)

Time period: Simulations were run for a period of 120 months, starting in Q1/2015.

**Results:** If all EPR-applying countries used EPR according to their legislation as of Q1/2015, prices would drop by on average 21.9% (base case) after 10 years. A consideration of discounts (assumed 20% discount in six large economies and the mandatory discount in Germany, Greece and Ireland) and a calculation of the reference price based on the lowest price in the country basket showed the highest impacts (-47.2% and -34.2% decreases compared to the base case). Adjusting medicine price data by purchasing power parities had a major impact on individual countries, leading to higher prices in some countries and lower prices in lower-income economies (overall drop by 16% compared to the base case). Regular price revisions, changes in the basket of reference countries and shorter intervals of the average exchange rate had also an impact, but to a lesser extent, on medicine prices.

**Conclusions and lessons learned:** The results showed that the methodological design of EPR can result in, partially substantial, changes in medicine prices. If EPR were mainly applied for cost-containment purposes, savings for the public payer could be obtained through strategic choices of the EPR methodology.

**Reference**

1. Schneider P, Vogler S. Practice of External Price Referencing (EPR). In: Vogler S, editor. Medicine Price Surveys, Analyses and Comparisons. London: Elsevier; 2019.

**Keywords:** Medicine prices, methodology, external price referencing, simulation, policy change

## ORAL PRESENTATIONS STRAND 3

### O10 Ten-year impact of the Access to Medicine Index: Changes in industry pricing and intellectual property policies in Low and Middle Income Countries from 2008-2018

#### Hans V Hogerzeil, Daniel J Edwards, Jayasree K Iyer

##### Access to Medicine Foundation, Amsterdam, The Netherlands

###### **Correspondence:** Hans V Hogerzeil (hans.hogerzeil@kpnmail.nl)

**Background:** The Access to Medicine (ATM) Index is a relative ranking of the performance of the world’s 20 largest research-based pharmaceutical companies in access to medicine in Low and Middle Income Countries (LMIC). The Index is published biennially since 2008. We performed a first longitudinal analysis of absolute progress made by these 20 companies during 2008-2018.

**Objectives:** Measure ten-year progress in pharmaceutical company policies and practices with regard to pricing and intellectual property policies in LMIC.

**Methodology:** Both public information and original data provided by the 20 companies for six ATM Indexes were re-analysed in a systematic approach to allow for longitudinal comparisons. Not all areas of analysis could be compared uniformly across all indices in this period.

Region covered: 106 Low- and Middle Income Countries covered by the Access to Medicine Index

Time period: 2008-2018

**Results:** During the study period, the number of companies with stated access to medicine policies rose from 8 to 17. The proportion of relevant products covered by equitable access strategies remained static at 33% in 2014 and 2016, and rose to 43% in 2018. The majority (53%) of the most robust pricing strategies (i.e. those focused on high disease burden countries, segmented within country, taking into account multiple factors to determine affordability) are concentrated in only three companies (Boehringer Ingelheim, Gilead, and Novartis). In terms of intellectual property (IP) policy, in 2018, 17 companies publicly disclosed patent information (up from none in 2008). In 2018, 29 compounds for HIV and hepatitis C were covered by voluntary licensing (12 in 2010). Little progress has been seen in public company endorsement of the Doha Declaration on TRIPS (Trade-Related Aspects of Intellectual Property Rights) and public health.

**Conclusions and lessons learned:** Companies are increasing efforts to develop access strategies, manage IP in an access-oriented manner, and consider affordability in LMIC, with notable shifts in patent transparency and licensing. Pricing strategies improve more slowly, with the most sophisticated applied by only very few companies. Companies fail to make complete public endorsement of the Doha Declaration.

**Keywords:** access to medicine index, pharmaceutical companies, low- and middle-income countries, pricing policies, intellectual property

### O11 Robust 5-year-forecast of the Austrian reimbursable pharmaceutical retail market

#### Karin Komposch^1^, Johannes Mertl^1^, Stefan Baumgartner^1^, Claus Burger^2^, Ulrich Lübcke^2^, Silvia Bauernhofer^3^, Wolfgang Trattner^4^, Martin Spatz^1^

##### ^1^IQVIA, Stella-Klein-Löw-Weg 15, 1020 Vienna, Austria; ²FOPI – Forum der forschenden pharmazeutischen Industrie, Vienna, Austria; ³Pharmig – Verband der pharmazeutischen Industrie Österreichs, Vienna, Austria; ^4^Österreichischer Apothekerverband – Interessenvertretung der selbständigen Apotheker, Vienna, Austria

###### **Correspondence:** Karin Komposch (karin.komposch@iqvia.com)

**Background:** In Austria, there is lack of availability of a Horizon Scan on the development of pharmaceutical spending essential for short, mid & long-term planning for stakeholders in the healthcare sector.

**Objectives:** Aim of the project is the annual generation of a fact-based 5-year-forecast of the Austrian reimbursable pharmaceutical retail market via involvement of multiple stakeholders that serves as common base for discussions – especially with payers – on pharmaceutical spending and pricing.

**Methodology:** The 5-year-forecast of the Austrian reimbursable pharmaceutical retail market is based on IQVIA databases that reflect sales of pharmaceutical products in the retail market. The term ‘reimbursable’ excludes products that are generally not covered by the Austrian Social Security Fund. Basis for the quantitative forecast are time series forecasts on historical data. For products with annual sales above EUR 2 million manufacturer (MNF) price (= focus products) forecasts are conducted at product level based on volume and rated at MNF price. For pharmaceutical products below this threshold, forecasting is performed at ATC-3-class level based on sales at MNF price and displayed as below and above co-payment fee. Focus product forecasts are attributed with qualitative information: Simulation of entry of generics and biosimilars is based on average uptake of historical generics and biosimilars launches. Pricing follows the generics and biosimilars pricing rule of the ASVG law. Forecast of focus products of 10 top pharmaceutical companies is validated by company representatives against company forecasts and R&D pipelines are rated for their annual sales potential. Average historical annual sales volume of new launches and average growth thereof is assumed as future product launch volume. Retrospective validation of the actual reimbursed volume of focus products was performed in cooperation with the Austrian Pharmacists Association.

Region covered: The 5-year-forecast covers the Austrian reimbursable pharmaceutical retail market.

Time period: The 5-year-forecast reflects the time period until 2023.

**Results:** The main result shows the average growth of the Austrian reimbursable pharmaceutical retail market until 2023 per reimbursement status. Furthermore, results show the share covered by the Austrian Social Security Fund set against the out-of-pocket private share of the Austrian population. Annual effects of price mechanisms that arise from the generics and biosimilars pricing rules are set against the impact of innovations.

**Conclusions and lessons learned:** The 5-year-forecast of the Austrian reimbursable pharmaceutical retail market developed with support of multiple stakeholders provides solid basis for fact-based discussions on pharmaceutical spending and pricing. Main future objective is to build up this forecast annually in cooperation with all relevant stakeholders.

**Funding Source**

Pharmig – Verband der pharmazeutischen Industrie Österreichs, FOPI – Forum der forschenden pharmazeutischen Industrie, FCIO – Fachverband der Chemischen Industrie Österreichs, Österreichischer Apothekerverband – Interessenvertretung der selbständigen Apotheker

**Keywords:** 5-year-forecast; Austrian reimbursable pharmaceutical retail market

### O12 Together working to improve access to medicines: analysis of cross-country collaborations in Europe

#### Rianne van den Ham^1^, Sabine Vogler^2^, Manuel Alexander Haasis^2^, Fatima Suleman^3^

##### ^1^WHO Collaborating Centre for Pharmaceutical Policy and Regulation, Utrecht University, Utrecht, The Netherlands; ²WHO Collaborating Centre for Pharmaceutical Pricing and Reimbursement Policies, Pharmacoeconomics Department, Gesundheit Österreich GmbH (GÖG / Austrian Public Health Institute), Vienna, Austria; ³WHO Collaborating Centre for Pharmaceutical Policy and Evidence Based Practice, University of KwaZulu-Natal, Durban, South Africa

###### **Correspondence:** Rianne van den Ham (h.a.vandenham@uu.nl)

**Background:** In recent years some cross-country collaborations of public authorities for pharmaceutical pricing and reimbursement were established to ensure affordable access to (high-priced) medicines.

**Objectives:** The study aims to identify and understand selected existing cross-country collaborations in the WHO European Region, including their intent and objectives as well as to assess their performance and analyse facilitating and challenging factors.

**Methodology:** Five European cross-country collaborations were selected: Baltic Procurement initiative (Estonia, Latvia, Lithuania), Beneluxa initiative (Belgium, the Netherlands, Luxembourg, Austria, Ireland), Nordic Pharmaceutical Forum (NPF: Denmark, Norway, Sweden and Iceland), Valletta Declaration (Croatia, Cyprus, Greece, Ireland, Italy, Malta, Portugal, Romania, Slovenia, Spain) and Visegrad collaboration (Czech Republic, Hungary, Poland, Slovakia). In addition to a literature and documents review, semi-structured interviews were held with representatives involved in the collaborations. A total of 19 interviews with 26 interviewees took place between July and October 2018. Using an analysis matrix, responses were examined with a view to exploring overarching patterns.

Region covered: WHO European region

Time period: Q2-Q3/2018

**Results:** In most cases, there was one country that led the initiative to form a collaboration. Four of the studied collaborations are political ones, with strong engagement at high political levels, whereas the NPF is a bottom-up initiative of technical experts. Three of the collaborations aim at performing joint price and/or reimbursement negotiations while joint procurement is included in the mission of the Baltic Procurement Initiative (procurement limited to vaccines) and the NPF. Cooperation in health technology assessment and horizon scanning form further activities in most of the studied initiatives, and the importance of information sharing has been stressed by all collaborations. Since most collaborations were rather new, ‘tangible results’ (e.g. joint procurements, joint negotiations) were not yet available. It is thus hard to assess the performance of the collaborations in terms of endpoints and efficiency. Nonetheless, officials involved in the collaborations clearly considered them as ‘success’ or ‘work towards success’. Facilitating factors include trust between the members, strong commitment of highly qualified technical experts, political backing, a structure within which to work (procedural rules) and information technology (e.g. videoconferences).

**Conclusions and lessons learned:** Information sharing is considered as a major value of the collaborations. Interviewees advised further governments to join existing collaborations or set up their cross-country cooperation. However, the starting phase is challenging, and it takes some time until the collaborations will be able to produce deliverables that are also regarded as successes by those not involved.

**Funding Source**

World Health Organization (WHO), Regional Office for Europe

**Keywords:** cooperation, joint procurement, joint negotiation, HTA, information sharing

### O13 Oncology drug market: a high-growth, high-price therapeutic area

#### Tanya Potashnik, Elena Lungu

##### Patented Medicine Prices Review Board, Policy Development, Ottawa, Canada

###### **Correspondence:** Tanya Potashnik (tanya.potashnik@pmprb-cepmb.gc.ca)

**Background:** Drug development has been dominated in recent years by oncology products, promising hope to patients and clinicians seeking access to medication for the fatal disease. The increased need for cancer products is turning oncology into a high-growth, high-price therapeutic area, fueled by the inflow of new launches with price tags that are continually reaching new highs.

**Objectives:** This presentation will provide decision makers, researchers, and patients with valuable insight into the dynamics of the oncology market from a Canadian and international perspective.

**Methodology:** The study reviews oncology drug approvals from Health Canada, the FDA, and the EMA and analyzes pricing and sales data from IQVIA’s MIDAS™ Database to examine the trends in availability, pricing, and sales in Canadian and international oncology markets, and to highlight major cost drivers.

Region covered: International markets examined include the countries in the Organisation for Economic Co-operation and Development (OECD), highlighting Canada and its comparator markets.

Time period: The analysis focuses on 2018, with retrospective trends back to 2009.

**Results:** International price comparisons show that Canada pays some of the highest prices for oncology medicines, though many major markets have greater availability. Oncology is a top driver of pharmaceutical spending in Canada, with sales nearly tripling over the last decade. Average treatment costs have almost doubled, and medicines with 28-day treatment costs over $10,000 now represent one third of total sales. Limited available therapeutic alternatives and longer market exclusivity have further exacerbated these cost pressures, as many oncology medicines are targeted, often biologic, therapies facing limited and delayed competition.

**Conclusions and lessons learned:** This analysis responds to a growing need to better understand and document the evolving oncology market, and provides decision makers, researchers, and patients with valuable insight into relevant market dynamics from a Canadian and international perspective.

**Funding Source**

Government of Canada

**Keywords:** Oncology, high-cost drugs, Canada

### O14 Supporting Decision-Making on Costly Hospital Medicines in Austria: Approaches for improved reimbursement processes?

#### Sarah Wolf, Claudia Wild

##### Ludwig Boltzmann Institute for Health Technology Assessment, Vienna, Austria

###### **Correspondence:** Sarah Wolf (Sarah.Wolf@hta.lbg.ac.at)

**Background:** The majority of western healthcare systems are confronted with limited healthcare resources and high healthcare expenditures, especially in the areas of orphan diseases and oncology. Thus, the introduction of new and ever more costly medicines requires decisions on a prioritisation of existing and new treatment options. All western countries have therefore implemented more or less transparent and standardised processes and methodologies to support such challenging decisions. In Austria, standardised processes for national reimbursement decisions are only in place for the outpatient sector, while reimbursement decisions on hospitals medicines are decentralised. This can result in unequal availability of and access to high-priced medicines between the nine Austrian federal states.

**Objectives:** The aim of the present study was to develop options for a national process for the reimbursement of costly medicines provided in the inpatient sector.

**Methodology:** Following a multi-stage approach, firstly the reimbursement processes of eleven countries including Austria were investigated. Secondly, the strengths and weaknesses of the elaborated options in the different procedural steps were analysed based on four criteria. Thirdly, three optional models of good practice for improvements for the Austrian reimbursement processes on inpatient medicines are suggested.

**Results:** Three optional models of good practice for improvements for the Austrian reimbursement processes on inpatient medicines were developed. The first option includes a reimbursement process for hospital medicines following the existing national reimbursement process of the outpatient sector in Austria. The second option represents a stronger coordination and cooperation of the nine regional “Pharmaceutical and Therapeutics Committees”. The third option illustrates an adaptation of the existing reimbursement process for non-pharmaceutical highly specialised technological interventions in Austria.

**Conclusions and lessons learned:** Evidence-based, transparent, fair and efficient resource allocations are cornerstones for the legitimacy of decisions in democracies. However, those four criteria can also be diametrically opposed: on the one hand, decision processes can be based on the best available evidence, can be fair in terms of involving various stakeholders and transparent in terms of public availability of information, on the other hand, it might be substantially more time-consuming. Thus, a pragmatic balance between timeliness, quality and transparency is crucial.

**Reference**

Wolf S, Wild C. Preisbildung und Arzneimittelerstattung im stationären Sektor in Österreich: Ansätze für einen transparenten und evidenzbasierten Prozess unter Berücksichtigung internationaler Erfahrungen. LBI-HTA Projektbericht Nr.: 109; 2018. Wien: Ludwig Boltzmann Institut für Health Technology Assessment.

**Keywords:** reimbursement process, national process, hospital drugs, democratic decision-making

## POSTER PRESENTATIONS STRAND 1

### P1 A comprehensive framework of pharmaceutical policies for decision-makers: functions, tools and data requirements

#### Ioana Ursu^1^, Viktoria Rabovskaja^2^

##### ^1^Mapping Health Limited, London, UK; ²GIZ GmbH, Deutsche Gesellschaft für internationale Zusammenarbeit, Eschborn, Germany

###### **Correspondence:** Ioana Ursu (i.ursu@mappinghealth.org)

**Background:** The World Health Report 2010 suggests medicines account for all three leading sources of inefficiency in health systems. Moving towards UHC, countries face the impact of these inefficiencies on their (new) health financing systems. Internationally, various policy solutions have been developed (e.g. formularies, HTA, pricing regulation etc). However, there is limited guidance for LMIC decision-makers on which intervention, when and how to adapt to their context.

**Objectives:** Develop a comprehensive framework encompassing the multitude of policies and tools that form a pharmaceutical system. The framework is conceived as an instrument for decision-makers to evaluate their current system, identify functional gaps, and choose reform interventions fitting their needs.

**Methodology:** The framework is the result of a multi-year, mixed methods work in public and private sectors. It is based on desk-review of pharmaceutical policies, HTA assessments and multiple rounds of qualitative interviews in 72 countries. The framework initially identified communalities of high resource systems, and then adjusted for middle-income settings (Eastern Europe). The framework was finalised and validated through qualitative research in Sub-Saharan Africa and South East Asian countries. The proposed framework addresses in- and out-patient settings across public and private sectors.

**Results:** The framework addresses three levels: 1) The building blocks (functions) of a pharmaceutical system (Figure 1); 2) The sequence of the functions and data flows among them 3) The interventions/types of data used or generated, per function (Figure 2). The framework has been recently used in Indonesia, Philippines and Togo. In Indonesia, it identified the main drivers behind the persistent out of pocket spending despite the newly introduced social health insurance. As result, rather than increasing effort to enhance the HTA, Indonesian decision makers considered a review of the prescribing patterns and the pricing methodology. In Philippines, the framework was used to create and integrate the HTA unit within Department of Health. It is also the basis for the development of the primary care benefit package under the recently signed UHC Act. In Togo, the framework helped develop a sustainable formulary for the public health insurance. The savings have been since planned to increase coverage for health emergency services.

**Conclusions and lessons learned:** The novelty of the framework consists of its systematic step-by-step guidance covering the pharmaceutical policy field. The framework can be applied across all settings: low, middle and high-income. It helps decision-makers and technical staff understand and envisage how the pharmaceutical system could be improved given the local context, timelines and capacity.

**Funding Source**

Various: GIZ, WHO, EU DEVCO The framework was developed in the course of multiple projects analysing and implementing pharmaceutical policies.

**Keywords:** Medicines, access, policy, pricing, HTA

**Fig. 1 (abstract P1).**
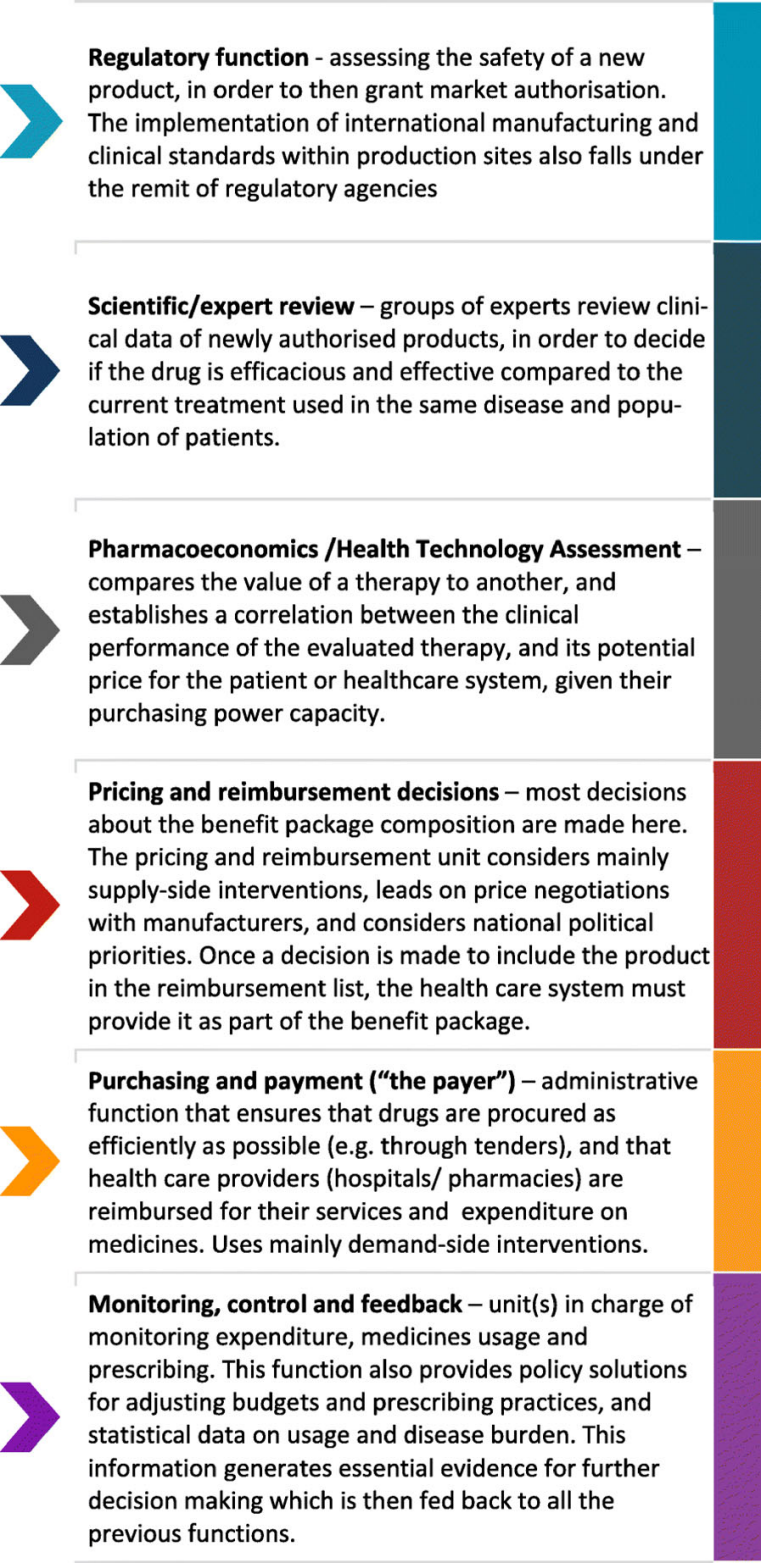
Mandatory functions of a pharmaceutical system

**Fig. 2 (abstract P1).**
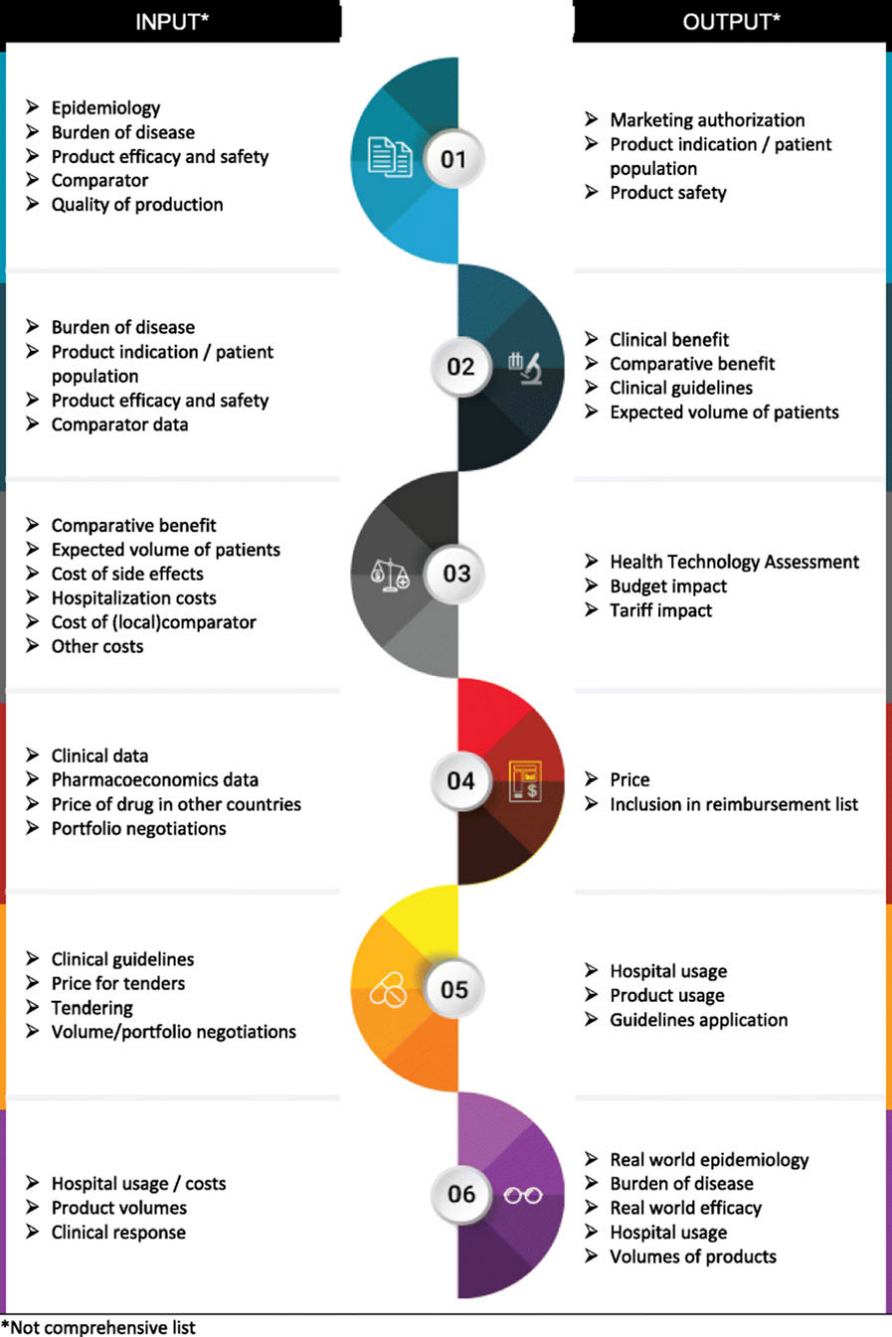
Interventions/ data-sets used and generated by each function

### P2 Drug Governance in the Emilia-Romagna Region, Italy

#### Francesco Nonino^1^, Maria Chiara Silvani^1^, Roberta Giroldini^1^, Elisabetta Pasi^1^, Lucia Magnano^1^, Giulio Formoso^2^, Anna Maria Marata^1^

##### ^1^WHO Collaborating Centre in Evidence-Based Research Synthesis and Guideline Development – Direzione Generale Curadella Persona Salute e Welfare, Servizio Assistenza Territoriale, Area Farmaco e Dispositivi Medici, Regione Emilia Romagna, Bologna, Italy; ^2^AUSL di Reggio Emilia, Reggio Emilia, Italy

###### **Correspondence:** Francesco Nonino (Francesco.Nonino@regione.emilia-romagna.it)

**Background:** The Italian public National Health Service provides assessment, pricing and reimbursement of medicines through the Italian Medicines Agency (AIFA). Although the reimbursement price is negotiated at a national level, each Italian Region implements its own drug governance policy adjusting it to local needs. The Emilia-Romagna Region (RER) adopted a policy relying on evidence-based recommendations on the use of medicines agreed with health professionals and patients. A Regional Drug Formulary (RDF) is produced and monthly updated by a Regional Drug and Therapeutic Committee (R-DTC) supported by multi-stakeholder workgroups (MWGs) with specific competence in different medical specialty areas. Medicines are purchased through centralized procurement procedures by a public independent regional agency.

**Objectives:** To develop a RDF as a tool to implement a sustainable, dependable and evidence-based medicine governance policy throughout the healthcare system of the RER.

**Methodology:** Guidance on the use of individual drugs or drug classes is prioritized according to unmet needs, efficacy, safety and economic impact. The recommendations issued by the R-DTC are informed by MWGs including health professionals, administrators and patients’ representatives. Evidence is systematically searched, appraised and summarized by a scientific secretariat. Guidance is produced as recommendations or regional guidance documents according to systematic and explicit methodology, such as the GRADE method [1]. Whenever feasible, cost-opportunity evaluations to foster competition among pharmaceutical companies are considered as a strategy of drug expenditure governance. In order to combine appropriate drug use, equitable access to healthcare and economic sustainability, quantitative indicators on expected prescription rates are provided with each recommendation. Comparisons between expected and observed prescription rates are regularly shared with clinicians and administrators.

Region covered: RER, Italy, 4-million inhabitants.

Time period: from 2006 to 2019.

**Results:** R-DTC updates the RDF monthly, with newly marketed drugs or new indications. At present RDF includes 1,242 drugs. Of the 255 documents issued by the R-DTC, 79 include evidence-based recommendations, 62 of which are graded according to the GRADE methodology [1]. To-date, 12 workgroups are active on the following topics: onco-hematology; biologic drugs in dermatology, rheumatology and gastroenterology; newer hepatitis-C drugs; cardiovascular and neurological conditions; diabetes; chronic renal impairment. Differences between observed and expected prescription rates were useful to understand the determinants of variability among prescribers and to inform decisions about resource allocation.

**Conclusions and lessons learned:** An explicit, transparent and flexible evidence-informed decision making process involving MWGs may allow more equitable access to treatments within a sustainable reimbursement system in a public health service.

**Funding Source**

Direzione Generale Cura della Persona Salute e Welfare, Servizio Assistenza Territoriale - Area Farmaco e Dispositivi Medici, Regione Emilia Romagna, Bologna, Italy.

**Reference**

1. Atkins D, et al; GRADE Working Group. Grading quality of evidence and strength of recommendations. BMJ. 2004 Jun 19;328(7454):1490.

**Keywords:** Drug Formulary, Therapeutic Committee, guidance, recommendation, GRADE method.

### P3 Mechanism for introduction of outpatient medicines in the reimbursement list in the Republic of Moldova: development and challenges

#### Rita Seicas, Ghenadie Turcanu, Stela Bivol

##### PAS Center, Chisinau, Republic of Moldova

###### **Correspondence:** Rita Seicas (rita.seicas@pas.md)

**Background:** In the context of global commitments to ensure extensive access to safe, effective, quality and affordable medicines, the assessment identifies barriers and factors that facilitate access to reimbursed medicines in the Republic of Moldova.

**Objectives:** The operational research of the national regulatory framework on developing the list of reimbursed outpatient medicines (LROM) by the mandatory health insurance funds aimed at identifying deficiencies and designing solutions for ensuring a transparent, holistic and feasible mechanism.

**Methodology:** The study had two components: 1) Analysis of the regulatory framework for outpatients medicines to be included in the LROM. 2) Qualitative research of the opinions and perceptions of the beneficiaries of medicines and actors of the system. Data sources: regulatory documents, reports published by the National Health Insurance Company (NHIC), qualitative data collected based on five focus groups and 33 in-depth interviews.

Region covered: National level (Republic of Moldova), WHO EURO

Time period: 01.01.2018- 30.05.2019

**Results:** Mandatory health insurance implemented in the Republic of Moldova has shown to be an effective tool for improving the population's access to medicines. Thus, starting in 2005, the benefit package included partial or full reimbursement of outpatient medicines. The LROM has evolved from 5 INN in 2005 to 148 INN in 2019. Public expenditures for reimbursed medicines increased from 7403.5 thousand Moldavian lei (MDL) in 2005 to 523 859.3 thousand MDL in 2017 [1]. At the same time, the LROM did not significantly change if compared to the national list of essential medicines. The first regulation on mechanism for introduction of outpatient medicines in the LROM was approved in 2010 and was revised fundamentally two times, with the most recent revision being done in 2015. The regulation included the cost-effectiveness criteria and evidence-based assessment methodologies; transparency; establishment of a technical secretariat to conduct the assessment. However, the Regulation is in need of further revision to: (1) improve transparency in establishing priorities for reimbursement; (2) re-introduce mandatory the cost–effectiveness criteria and budget impact analysis, since the revision of 2017 made them optional; (3) develop guidelines to enhance coherence and justifications of the process; (4) involve multidisciplinary expert teams [2]. Qualitative research highlighted that access to LROM is perceived differently by different categories of population and actors of the system.

**Conclusions and lessons learned:** Substantial steps have been taken to improve the mechanism of developing the LROM, but further efforts will be need to be undertaken to achieve long-lasting changes in the area of transparency, relevance of decisions, revisability, and implementation.

**References**

1. NHIC, Annual activity report for 2017.

2. MoH and NHIC Order # 600/320 as of 24.07.2015.

**Keywords:** Reimbursement outpatient medicines, regulation, focus group, consumer’s perception

### P4 Case study of the judicialisation of eculizumab (Soliris®): challenges in the price regulation and the impact of establishment of the maximum government price in Brazil

#### Adriana M Ivama-Brummell^1^, Juliana A Ortiz^1^, Daniella Pingret ^1^, Leandro P Safatle^2^

##### ^1^Medicines’ Market Regulation Chamber Executive Secretariat (SCMED)/Brazilian Health Regulatory Agency (Anvisa), Brasília, Brazil; ²Direb/Oswaldo Cruz Foundation (Fiocruz), Brasília, Brazil

###### **Correspondence:** Adriana M Ivama-Brummell (adriana.ivama@anvisa.gov.br)

**Background:** In Brazil, access to health, including the access to medicines is a Constitutional right to be fulfilled through public policies in the Brazilian Unified Health System (SUS). Due to limited budgets, the “*judicialisation*” (court cases) has been a strategy used by individual patients to fulfil their rights when the system fails to provide for them.

**Objectives:** The objective of this study was to describe and review how the economic regulation has been contributing to promote access to medicines for very high-priced medicines in Brazil.

**Methodology:** A policy analysis was conducted combining a descriptive study with data review from the Monitoring System of Medicines (SAMMED) and the national public procurement system (Compras-net) regarding the procurement of eculizumab (Soliris**®**) from 2010 to 2018, reviewing key results.

Region covered: This national study was carried out in Brazil (PAHO/WHO region).

Time period**:** November 2018 to April 2019

**Results:** The Medicines’ Market Regulatory Chamber (CMED) regulates medicines’ prices (price cap), based on Health Technology Assessment, External Reference Pricing (ERP) and Internal Reference Pricing (IRP). In 2006, CMED established the Price Acquisition Coefficient (CAP), a mandatory minimum discount with a maximum government procurement price (PMVG) to a positive list of medicines. In 2016, eculizumab (Soliris**®**), for treatment of paroxysmal nocturnal haemoglobinuria (PNH), a rare disease, costed USD 187 million (R$ 620 million) to the SUS (average unit price: USD 8.347,82, R$ 27,614.60), purchased due to court cases, before marketing authorisation and its incorporation to the health system. In 2017, with the CAP discount of 19.28%, CMED established the PMVG of USD 3.710,00 (R$ 12,274.83). Due to this, in 2018, the Ministry of Health (MoH) purchased more than twice the volume (31,056 units for 431 patients) compared to 2017 (13,721 units for 190 patients), based on the recommended daily doses for adults in the main indication (Figure 1).

**Conclusions and lessons learned:** Despite great savings, contributing to increase the access to medicines, there are still challenges, limitations and sustainability risks for the health system in providing very high-priced medicines, with few or none external reference prices. The quick launching of a medicine in several countries can push the prices upwards for countries using ERP as in Brazil, where there are no provisions for reviewing prices when new evidences appear. As lessons learnt: the legal provision for setting a provisional maximum price and PMVG “*ex oficio*” with administrative process and penalties for commercialisation before approval can prevent abusive prices.

**Keywords:** high price medicines, economic medicines regulation, external reference pricing, value-based pricing, pharmaceutical policies

**Fig. 1 (abstract P4).**
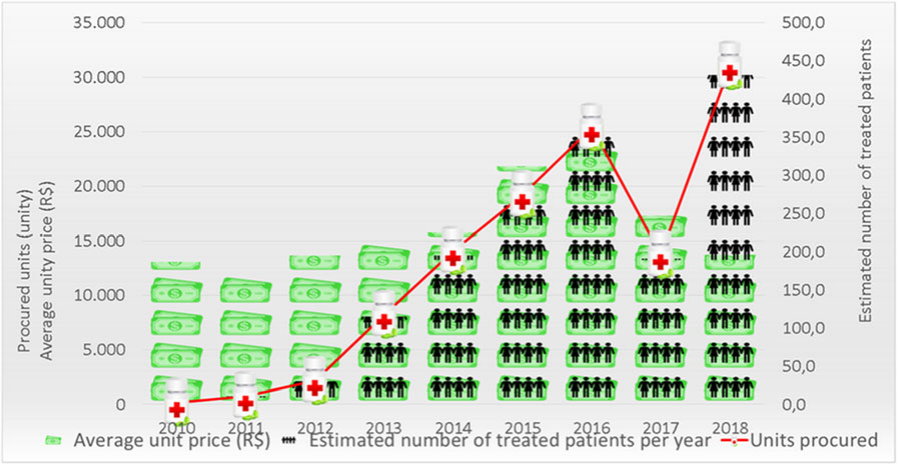
Number of units, average unit price of eculizumab (Soliris**®**) procured by the Brazilian Ministry of Health and the estimated number of treated patients from 2010-2018. * The average procurement price was calculated based in the different procurement processes through each year and number of units and the estimated number of treated patients per year was calculated based in the adults’ recommended daily doses for paroxysmal nocturnal haemoglobinuria (PNH)

### P5 18 years of economic regulation of medicines in Brazil: outcomes, challenges and lessons learnt

#### Adriana M Ivama-Brummell^1^, Juliana A Ortiz^1^, Daniella Pingret^1^, Rosiene R de Andrade^1^, J Ricardo Santana^1^, Leandro P Safatle^2^

##### ^1^Medicines’ Market Regulation Chamber Executive Secretariat (SCMED)/Brazilian Health Regulatory Agency (Anvisa), Brasília, Brazil; ²Oswaldo Cruz Foundation (Fiocruz), Brasília, Brazil

###### **Correspondence:** Adriana M Ivama-Brummell (adriana.ivama@gmail.com)

**Background:** The end of last century in Brazil was a turning point of an immense crisis in the pharmaceutical sector, with falsified and substandard medicines, shortages, very high prices, among other practices, towards the approval of national policies and new economic and health regulatory frameworks for medicines, including the establishment of the Medicines’ Market Regulation Chamber (CMED).

**Objectives:** to review the implementation of the economic regulatory framework for medicines in Brazil and the adopted regulatory policy options based on WHO recommendations, describing its outcomes, challenges and perspectives.

**Methodology:** A policy analysis was conducted combining descriptive with qualitative analysis with official data review from the Medicines’ Market Monitoring System (SAMMED) and the national public procurement system (Compras-net).

Region covered: This national study was carried out in Brazil (at PAHO/WHO region).

Time period**:** November 2018 to April 2019

**Results:** Preliminary results show that the national policies and the regulatory framework following WHO recommendations (Table 1) provided for a stable structure and governance mechanisms, with a technical body to support decision making, a monitoring system and enforcement, leading to medicines’ price stability, arising mostly below inflation levels (Figure 1). The policy interventions included (Table 2): price cap based on health technology assessment (HTA), external reference pricing (ERP) and internal reference pricing (IRP); generic medicines at 65% of the reference medicine prices; mandatory discounts for public procurement (PMVG); annual prices adjustments; tax exemptions; updated electronic price lists publicly available online; monitoring of the pharmaceutical market with mandatory reports of commercialisation data with mechanisms of compliance and enforcement. From 2011 to 2017, 230 new medicines entered the Brazilian market. 201 (87%) of them from transnational and 29 (13%) from national companies, with 25 different therapeutic classes. In 2017, the revenue of the Brazilian pharmaceutical market was USD 21 billion with 4.4 billion units commercialised (1.4 billion units of generic medicines, 32.4%). In 2018, the mandatory discount for public procurement was 20.16% of the maximum prices, leading to important savings.

**Conclusions and lessons learned:** Predictability and transparency were key for ensuring price stability. The intersectoral governance mechanism of CMED contributed for its consolidation as part of a State policy, which continued through different governments. The pharmaceutical sector continued growing, even during austerity periods. The challenges include the need of improving the regulatory framework, appraisal process and transparency and finding alternatives for high priced medicines with preliminary or poor-quality evidence and without ERP.

**Keywords:** economic medicines regulation, external reference pricing, internal reference pricing, value-based regulation, health technology assessment

**Fig. 1 (abstract P5).**
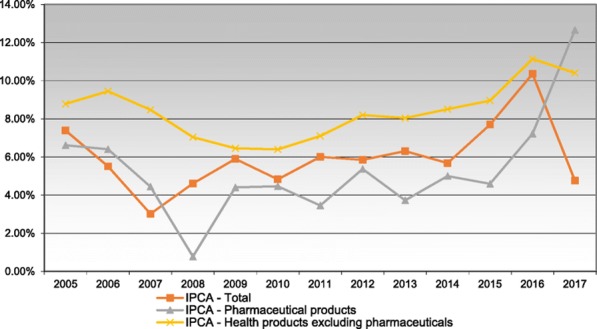
IPCA (Broad Consumer’s Prices National Index) and average authorised adjustment of the medicines’ market (2005 - 2017). Source: SCMED

**Table 1 (abstract P5).** Summary of the principles recommended by WHO and implemented in Brazil

WHO recommended principles	Implemented policy intervention/outcome*
Countries should use a combination of different pharmaceutical pricing policies that should be selected based on the objective, context and health system.	Brazil has a combination of different legal instruments establishing pharmaceutical pricing policies, including medicines production and innovation, the regulation of medicines prices, incorporation and procurement established by different laws and policies.
Countries should make their pricing policies, processes, and decisions transparent.	The Law 10.742/2003, Resolution CMED 02/2004 and additional regulations establish the criteria for pricing. The decisions taken are transparent as they are publicly available. Guidelines for the technical report for price decisions is under development.
Pricing policies should have an appropriate legislative framework and governance and administrative structures, supported by technical capacity, and should be regularly reviewed, monitored (including actual prices) and evaluated and amended as necessary.	The Law 10.742/2003 sets the basis for medicines prices regulation and established a governance and administrative structure – the Medicines’ Market Regulatory Chamber (CMED) a cross-government body with representatives from the Ministry of Health (President), the Presidency’s Office (Casa Civil), the Ministry of Economy and Ministry of Justice and Public Security and its Executive Secretariat at the Brazilian Health Regulatory Agency (Anvisa). The decision-making levels are the Ministerial Council, the Executive Technical Committee (CTE) and its Executive Secretariat (SCMED), a technical body for supporting the decision making, implementing its decisions and monitoring the pharmaceutical market. The price decisions as well as the approved prices are publicly available.
In promoting the use of affordable medicines, countries should employ a combination of pharmaceutical policies that address both supply and demand issues.	The national medicines policy (1998), pharmaceutical services policy (2004) and science and technology in health policy (2005) address both supply and demand issues. These policies were formally approved and implemented.
If regulation of pharmaceutical prices is introduced, effective implementation will be required to ensure compliance (e.g. incentives, enforcement, price monitoring system, fines).	As established by the Law 10.742/2003 and additional regulations, enforcement mechanisms, including a monitoring system with policy power are in place to ensure compliance of the price regulation. *Price adjustments:* Once the prices are set, annual adjustment are authorised (not mandatory) based on three main factors: productivity factor, intra-sectoral factor and inter-sectoral factor. All the parameters for calculating these factors are defined and publicly available and one of them is the Broad Consumer’s Prices National Index (IPCA).
Countries should adopt policies to promote the use of quality assured generic medicines in order to increase access and affordability.	Generic medicines’ policy and legal framework were established by the Law 9.787/1999 and have been fully implemented. Rules for pricing of generic medicines were established by the Resolution CMED 2/2004. Regulations from Anvisa set requirements for quality, safety, efficacy, prescribing by the international nonproprietary name (INN) and generic substitution.
Countries should collaborate to promote exchange of information about policies, their impacts, and pharmaceutical prices.	Brazil is a member of networks of the America’s Regional Initiative of competent authorities related to price policies and regulation and the network of Health Technology Assessment of Americas (Redetsa), both supported by PAHO/WHO.

*the list of described implemented policy interventions is not exhaustive. Measures implemented in the country by other stakeholders may not be fully acknowledged as were not in the scope of the study

**Table 2 (abstract P5).** Summary of the price regulation policy interventions recommended by WHO and implemented in Brazil

WHO recommended policy intervention	Implemented policy intervention/outcome*
Regulation of mark-ups in the pharmaceutical supply and distribution chain	The mark-up is obtained by the difference between the ex-factory (PF) and maximum consumer’s price (PMC). PF is the maximum price for manufacturers and PMC is the maximum retail price for pharmacies. They are established by the Interpretative guideline CMED nº 02/2006 and an average mark-up of 38% is allowed. Nevertheless, the margin from the manufacturer to the wholesaler is not regulated. This issue is currently subjected to research at the CMED.
Tax exemptions/ reductions for pharmaceutical products	There is exemption for the positive list of prescription medicines from the federal taxation on commercialisation. More than 70% of the medicines in the Brazilian market are exempt from Federal taxation (PIS/Cofins) on commercialisation, mostly, prescription medicines and 95% of the medicines that are not exempt are over the counter (OTC).
Application of cost-plus pricing formulae for pharmaceutical price setting	Cost-plus pricing is not used in Brazil.
Use of external reference pricing	External reference pricing is used to set the maximum prices (ex-factory, consumer price) in combination with Health Technology Assessment (HTA) for new/innovative medicines with added therapeutic benefit compared with the existing medicines available in the Brazilian market or new/non-innovative medicine without a comparator in the Brazilian market. Brazil uses a basket of 9 countries (Australia, Canada, Spain, United States of America, France, Greece, Italy, New Zealand and Portugal, plus the country of origin.
Promotion of use of generic medicines	Legislative measures for early market entry, prescription by INN and generic substitutions are in place. Price of generic medicines are set as 65% of the reference medicines. Prices are set using internal reference pricing (IRP). Lists of authorised prices of medicines (including generics) are publicly available
Use of health technology assessment	A legal framework is in place to define both mechanisms and institutions responsible for price setting as a mandatory requirement for commercialisation and incorporation of medicines in the Unique Health System (SUS), the Brazilian National Health System. HTA is used in price setting for new medicines in combination with ERP and IRP and the prices defined are publicly available. HTA is also used for decision making regarding incorporation of medicines to the SUS and decisions are publicly available.
Other policy interventions	*Mandatory discounts for public procurement* were established by CMED based on the ex-factory maximum price authorised, namely Price Adequation Coefficient (CAP). The resulting price is the Maximum Price for Sales to the Government (PMVG), which is updated annually, and it is currently 20.16%.

*the list of described implemented policy interventions is not exhaustive. Measures implemented in the country by other stakeholders may not be fully acknowledged as were not in the scope of the study

### P6 Pharmaceutical pricing and reimbursement policies – Comparative analysis in 47 PPRI member countries

#### Nina Zimmermann, Sabine Vogler, Margit Gombocz

##### WHO Collaborating Centre for Pharmaceutical Pricing and Reimbursement Policies, Pharmacoeconomics Department, Gesundheit Österreich GmbH (GÖG / Austrian Public Health Institute), Vienna, Austria

###### **Correspondence:** Nina Zimmermann (nina.zimmermann@goeg.at)

**Background:** To facilitate affordable and equitable access to essential and cost-effective medicines for patients, governments can use a mix of policy measures. For the implementation and optimisation of such policies, policy makers benefit from information and evidence of appropriate measures in other countries and their impacts.

**Objective**s: The study aims to provide a comprehensive, concise and up-to-date comparative analysis of pharmaceutical pricing and reimbursement policies implemented in the 47 member countries of the PPRI (Pharmaceutical Pricing and Reimbursement Information) network of competent authorities.

**Methodology:** Information and data of pharmaceutical policies, mainly in the area of pricing and reimbursement, in both outpatient and inpatient sectors were collected from several sources, however, primarily through primary surveys of competent authorities in 47 countries that are involved in the PPRI network.

Region covered: 44 countries of the WHO European Region plus Canada, South Africa and South Korea

Time period: December 2018 or the latest available year

**Results:** Almost all PPRI countries have mechanisms in place to set medicine prices at the ex-factory (or sometimes wholesale) price level, mostly targeting reimbursable medicines or prescription-only medicines. 41 of the PPRI countries apply external price referencing to derive a benchmark for setting national medicine prices, at least for parts of the medicines. Its methodology (e.g. reference countries, benchmark calculation) varies across the countries. Among the PPRI countries, Sweden is the only country with a fully-fledged value-based pricing system. In several other PPRI countries, health technology assessments (HTA) and pharmacoeconomic instruments are used to support mainly reimbursement decisions of new medicines. Several but not all PPRI countries have regulated distribution remuneration (e.g. mark-ups; 32 countries with regulated wholesale remuneration and 43 countries with regulated pharmacy remuneration). Almost all PPRI network member countries have one or more reimbursement lists for outpatient medicines in place. At least 42 countries apply co-payments for outpatient reimbursable medicines (frequently percentage co-payments, but also prescription fees and deductibles). The 42 countries apply exemptions from or reductions of co-payments for vulnerable groups.

**Conclusions and lessons learned:** Since the implementation of pricing and reimbursement policies is in the national competence of governments, policies used vary greatly with regard to their aims, design and enforcement. For identifying best-practice policies with regard to facilitating affordable and equitable access to essential and cost-effective medicines further research is needed.

**Funding Source**

Austrian Federal Ministry of Labour, Social Affairs, Health and Consumer Protection

**Keywords:** pharmaceutical policy, comparative analysis, pricing and reimbursement

### P7 The experience of the Tuscan Region in managing biosimilar penetration

#### Elisa Guidotti^1^, Bruna Vinci^1^, Francesco Attanasio^2^, Federico Vola^1^

##### ^1^Laboratorio Management e Sanità, Institute of Management and Department EMbeDS, Scuola Superiore Sant’Anna Pisa, Italy; ^2^Drugs and appropriateness policy sector, Tuscan Regional Authority, Florence, Italy

###### **Correspondence:** Elisa Guidotti (elisa.guidotti@santannapisa.it)

**Background:** Italy is a leading country in the uptake of biosimilars and their use has been constantly growing; however, their distribution is not uniform across Regions. Most Regions have implemented specific policies concerning biosimilar governance to guarantee equity and financial sustainability [1].

**Objectives:** Some Italian Regions established policies to promote the entry of biosimilars into the therapeutic plans (i.e. Tuscany, Emilia-Romagna); others have drawn up late and unfocused policies having a low penetration of biosimilars (i.e. Lazio, Sardinia) [1]. The purpose of this research was to investigate which governance tools support a high penetration of biosimilars ensuring equity and financial sustainability. The case of the Tuscany Region was analysed.

**Methodology:** Regional pharmaceutical administrative flows were analyzed to identify the penetration rate of biosimilars in Tuscany. Molecules with low penetration and high potential for economic savings were selected and a catalogue of indicators for these molecules realized. An engagement process with managers and specialists of Tuscan Local Health Authorities was started to discuss the indicators and define shared targets of increasing the uptake. The engagement process was soon transformed into regular meetings to monitor the achievements, benchmark against each other and revise objectives.

Region covered: The study, carried out at regional level, focused on the experience of Tuscany, a medium-sized Italian region of 3 736 968 inhabitants.

Time period**:** June 2019 - September 2019

**Results:** The panel of indicators on biosimilars, the definition and continuous revision of shared targets and the constant and systematic benchmarking fostered biosimilars penetration over the period 2017-2018 in Tuscany. The percentage of biosimilar molecule Etanercept, for instance, has grown from 21.05% to 68.69%, the % Biosimilar Rituximab from 7.1% to 74.64%. The increase has been either greater or in line with that of the other Italian regions. The greater usage of biosimilars has contributed to the reduction of the pharmaceutical expenditure of the Tuscan Region from EUR 1.157.044.094 in 2017 [2] to EUR 1.118.523.838 in 2018 [3]. However, both a significant intra-regional and inter-regional variability has been observed.

**Conclusions and lessons learned:** The set of governance actions implemented in the Tuscan Region led to a significant increase in the penetration of selected biosimilar molecules. The consequent economic savings allowed for available resources to be reinvested in new and promising molecules. However, biosimilar penetration still has room to increase and variability remains high. Thus, further governance actions should be undertaken to increase the uptake and reduce the variability.

**References**

1. Centro Studi IQVIA Italia. Farmaci biologici e biosimilari, Scenari terapeutici e stima del risparmio per il Sistema Sanitario Italiano. Milan: IQVIA Solutions Italy S.r.l.; 2018. 21-25.

2. Agenzia Italiana del Farmaco. Monitoraggio della Spesa Farmaceutica Nazionale e Regionale (gennaio / dicembre 2017 (Consuntivo)). Rome: AIFA; 2019. 38-39.

3. Agenzia Italiana del Farmaco. Monitoraggio della Spesa Farmaceutica Nazionale e Regionale (gennaio / dicembre 2018 (Primo Rilascio)). Rome: AIFA; 2019. 36-37.

**Keywords:** governance tools, biosimilar penetration, Tuscany, Italy

### P8 The rising costs of Orphan Drugs in Italy

#### Enrico Costa^1,2^, Paola Marini^1^, Massimo Riccaboni^3^, Claudio Jommi^4^

##### ^1^Department of Pharmacy, University Hospital of Verona, Italy; ^2^WHO Collaborating Centre for Pharmaceutical Policy and Regulation, Utrecht University, The Netherlands; ^3^AXES research unit IMT School for Advanced Studies, Lucca, Italy; ^4^Cergas, SDA Bocconi School of Management, Milan, Italy

###### **Correspondence:** Enrico Costa (enrico.costa@aovr.veneto.it)

**Background:** OMPs are drugs intended for the treatment of serious conditions affecting less than 5 in 10,000 people in the EU. Although the ‘orphan’ designation allows applicants to benefit from incentives and conditional marketing authorization by the EMA to sustain their development, OMPs are characterized by high prices affecting their access across Europe.

**Objectives:** This paper aims to give some insights into the Italian Pricing & Reimbursement (P&R) Policies on Orphan Medical Products (OMPs) highlighting the strengths and weaknesses of the system.

**Methodology:** Data on the Pharmaceutical Expenditure (PE), P&R procedures and the legal framework came from the National Report on Medicines use in Italy of the Italian Medicines Agency (AIFA).

Region covered: Italy

Time period: 2017

**Results:** In Italy the expenditure for OMPs increased from EUR 652 million in 2010 (3.5% of the whole public PE) to EUR 1,599 million in 2017 (7.2%). Some OMPs are ranking within the first 30 top-selling drugs. Out of the 99 OMPs authorized by the EMA, 85 were reimbursed by the AIFA. The remainders were either marketed though temporary not-reimbursed or accessible through law 326/2003 (AIFA 5% Fund), which provides the reimbursement of not-yet-marketed OMPs through a fund financed by the 5% of annual expenses for the promotion activities of the pharmaceutical companies. In 2017 the AIFA fund supported the access to 13 OMPs for 40 patients (EUR 13.465.742). AIFA may grant a medicine the status of innovative drug according to 3 criteria: unmet medical needs, clinical added value and quality of evidence. This allows access to special funds, exemption from payback mechanisms and the immediate availability at local/regional level. Unlike non-orphan drugs - where high-quality of evidences are required - OMPs may be granted the status of innovative also when the level of evidence is moderate or low.

**Conclusions and lessons learned:** In Italy the policies on OMPs are largely inclusive: the NHS allows the access to these drugs even before standard marketing authorization through special pathways. Although these procedures are limited to patients affected by life-threatening or debilitating conditions without any therapeutic alternatives, the high prices and the increasing number of OMPs marketed every year have been weakening the sustainability of the healthcare system. Incentives provided at EU level, along with the status of innovative granted by the AIFA – even in presence of moderate or low level of evidence - were set up to sustain the survival of OMPs, not to make them the new blockbusters.

**Reference**

The Medicines Utilisation Monitoring Centre. National Report on Medicines use in Italy. Year 2017. Rome: Italian Medicines Agency, 2018. Available from: www.aifa.gov.it

**Keywords:** Orphan drugs, rare diseases, affordability

### P9 Biosimilar uptake in Denmark- A review of success

#### Dorthe Bartels, Trine Ann Behnk

##### Amgros I/S; Kopenhagen, Denmark

###### **Correspondence:** Dorthe Bartels (dbs@amgros.dk)

**Background:** In Denmark, hospital drug prices are the result of a national tendering process performed by the procurement body AMGROS I/S. Mechanisms in the market include analogue competition and a strategic procurement system. The evolution of Biosimilar uptake in Denmark is often referred to as a “successful implementation of biosimilars”. An increase in requests from other countries on what is key to this success has led Amgros to perform an evaluation of the process and outcomes based on quantitative and qualitative data. This has led to a documentation of the learnings and a documentation of the process with a focus on how to ensure a successful biosimilar uptake.

**Objectives:** The aim of this paper is to document best practice and share recommendations based on the biosimilar success in Denmark in the past 3 years, using Etanercept (ETA), Infliximab (INF) and Adalimumab (ADA) as an example.

**Methodology**: The abstract is based on a single case study, using both quantitative register data as well as qualitative data from evaluations in each phase in the End to End process. It is focused on one single therapeutic area (ETA, INF, ADA) for the case study design using a combination on what we have seen over time. The single case study covers procurement in the public sector, in-patient sector, and in hospital pharmacies.

Region covered: Denmark, EU

Time period: Longitudinal data (2016-2019)

**Results:** The evaluation of uptakes in Denmark over the period 2015-2018 shows how the evolution impact happens with increasing focus on qualitative implementation. Figure 1 above shows that the importance of the task with introducing biosimilars triggers challenges to be overcome. Preparation and communication are vital.

Key learnings are that the integrated partnership and refining and improving each step in the planning and execution phase throughout the whole process were crucial proceedings for a successful uptake of biosimilars.

Even practical challenges needed to be recognised and individually handled, rather than dealt with on a generic level.

**Conclusions and lessons learned:** Amgros, as procurement body, developed together with other stakeholders a process for implementation of biosimilars.

This is shown in Figure 2: Planning, Dialogue, Involvement Flow.

The learnings have involved more elements, both on organisational structure and insight sharing as well as on the practical and logistical side after the procurement are finalized.

Specific learning elements are generic and are captured in this review of how a stepwise well -prepared process supports and maximize biosimilar uptake in a country.

**Funding Source**

Amgros I/S

**Keywords:** Biosimilars, procurement, best practice, task force, implementation technique

**Fig. 1 (abstract P9).**
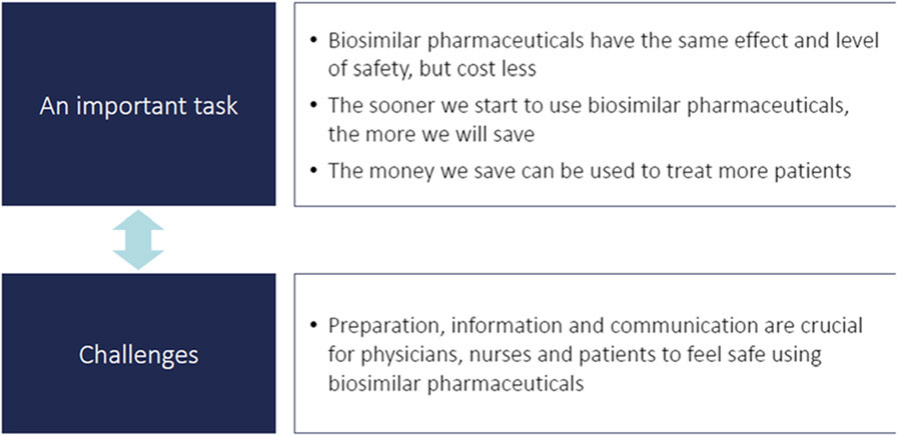
Preparation is vital

**Fig. 2 (abstract P9).**
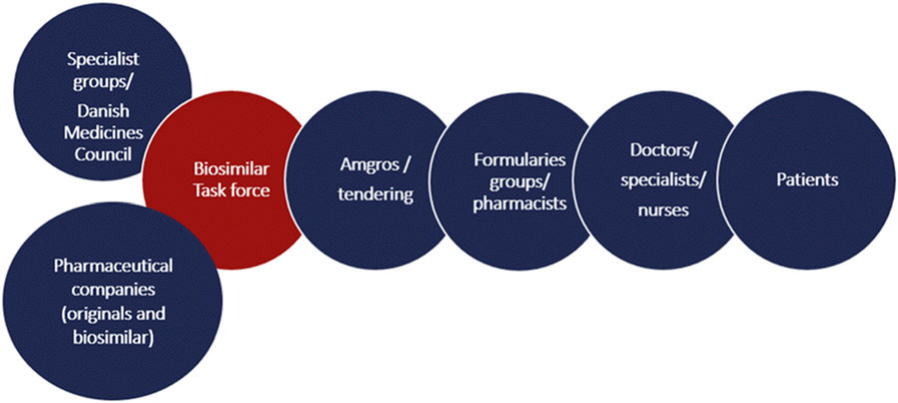
Planning, Dialogue and Involvement Flow

### P10 Insight into the Market for New Medicines

#### Tanya Potashnik, Elena Lungu

##### Patented Medicine Prices Review Board, Policy Development, Ottawa, Canada

###### **Correspondence:** Tanya Potashnik (tanya.potashnik@pmprb-cepmb.gc.ca)

**Background:** New medicines are a high-growth market segment in Canada, reaching almost a third of all pharmaceutical sales. High-cost specialty medicines are increasingly dominating the landscape, which include biologics, orphan drugs, and cancer products, have treatment costs in tens or hundreds of thousands of dollars per year.

**Objectives:** To provide an overview of emerging pharmaceuticals with the potential to impact healthcare in Canada, as well as to explore the market entry dynamics of new medicines in Canada and internationally.

**Methodology:** Capturing data from various sources, including the IQVIA MIDAS™ Database, FDA, EMA, and Health Canada, and GlobalData, this presentation explores the continuum of new medicines in the international market, monitoring potential candidates in late stages of clinical development and analyzing the market entry dynamics of those launched in Canada and internationally in 2018. Pipeline medicines are selected for their potential impact on future clinical practice and/or drug spending. New launches are assessed based on the date of first-time market approval through the US FDA, the European Medicines Agency (EMA), and/or Health Canada. The presentation analyzes the availability, sales, and pricing of new medicines in Canada compared to international markets.

Region covered: International markets examined include the countries in the Organisation for Economic Co-operation and Development (OECD), highlighting Canada and its comparator markets.

Time period: Pipeline medicines and new launches are assessed using 2018 data, with a retrospective analysis of trends since 2009.

**Results:** Of the 733 new medicines in late stages of clinical evaluation, 30 were selected for the pipeline study based on their potential impact on future clinical practice and/or drug spending in Canada. These medicines were drawn from a broad range of therapeutic areas, and include 20 medicines with orphan drug designations, nine oncology medicines, and three biologics. The high proportion of specialty medicines in the pipeline is also reflected among new launches; an international analysis finds that orphan medicines increasingly dominate the market, and cancer treatments represent over a quarter of new launches. Profiles of the new drug landscape in recent years suggest that high-cost drugs are becoming the norm rather than the exception for this market.

**Conclusions and lessons learned:** In analyzing the dynamics of both pre- and post-authorization markets, this study captures a unique picture of the impact of new medicines on drug expenditures, enabling policy-makers and stakeholders to better anticipate, manage and respond to evolving cost pressures and inform discussions on longer-term system sustainability.

**Funding Source**

Government of Canada

**Keywords:** New medicines, pipeline, oncology, orphan drugs, Canada

### P11 Good practice to improve the supply of hospital medicines and prevent backorders

#### Lars Erik Munck

##### Amgros I/S, Copenhagen, Denmark

###### **Correspondence:** Lars Erik Munck (lmu@amgros.dk)

**Background**: After years with an increasing number of backorders and many unplanned drug changes implemented under time pressure in the hospitals, we decided in 2017 to replace working in “firefighting mode” with proactiveness through better supply chain transparency between suppliers and hospital pharmacies (Tax funded, public sector).

**Objectives:** The aim is to improve the supply of medicines to hospitalised patients in Denmark and to reduce the increasing number of backorders from suppliers.

**Methodology:** We established a national Sales & Operations Planning (S&OP) unit, to develop and implement a national S&OP process for all medicines on national tenders. The S&OP process will ensure that hospital pharmacies estimate their demand of each item-number and that suppliers confirm their supply capability accordingly or report potential supply problems before stock-out. This S&OP planning process was established as an intervention with hospital pharmacies and suppliers. It consists of a combination of a qualitative and quantitative methods: 1) Involving Hospital Pharmacies and suppliers in the step-by-step process development. 2) Helping Hospital Pharmacies to estimate, and identify estimates that needs revision, to improve accuracy. 3) Active communication of estimates to suppliers. 4) Rebuilding supplier’s trust in our estimates as accuracy improved. 5) Asking suppliers to confirm supply capability. 6) Open and cross-functional dialogue about possible solutions to potential supply problems. Time period: 2017-2019

**Results:** The number of backorders in Denmark has stabilised during 2017-2019, whilst other countries have experienced a sharp backorder increase. Proactive solutions/decisions for potential supply problems have improved the overall supply situation/-information, and have improved patient safety, as fewer unplanned drug-changes are implemented under time pressure. Transparency across the supply chain has generated trust and enabled more value-adding and cross-functional dialogue e.g. sharing causes for estimate changes and early sharing of potential supply problems.

**Conclusions and lessons learned:** It’s hard work to implement a new focus area with many stakeholders, but be patient and focused, and results will show. Now we receive positive feedback from both hospital pharmacies and suppliers regarding resources/benefits from participating in the S&OP process.

Next steps: Assisting hospital pharmacies in getting input about future drug changes from hospitals. Understand how other countries are managing demand/supply and gather input to further improve our S&OP.

**Funding Source**

Amgros I/S

Keywords: Demand, supply, shortage, backorders, S&OP, supply chain transparency, drug changes

### P12 Analysing and controlling of Pharmaceutical Expenditures of National Health Insurance Fund, Sudan: Paying for value

#### Isam Eldin Ahmed

##### National Health Insurance Fund, Khartoum, Sudan

###### **Correspondence:** Isam Eldin Ahmed (pharmkal@hotmail.com)

**Background:** Inaccessibility to medicines is a common problem worldwide. The Pharmaceutical Expenditures represent 45% out of the total health expenditures of the National Health Insurance Fund (NHIF). In November 2016 the Central Bank of Sudan has Liberalized the exchange rate which increased from 1 USD = 6.5 Sudanese Pounds (SDG) to 1 USD = 15.8 SDG, that led the Regulatory Body to reprice the medicines. The Pharmaceutical Expenditures will exceed the total budget of the NHIF unless effective intervention based on deep cost analysis would be implemented.

**Objectives:** 1. To analyze the total cost of pharmaceuticals of NHIF in Sudan to find opportunity for cost reduction. 2. To identify the top ten costly medicines in comparison with the therapeutic benefits. 3. To select the most cost-effective interventions to contain the cost and improve the use of medicines

**Methodology:** A total cost analysis was performed using ABC, VEN and Therapeutic Categories tools. The Pharmaceuticals purchased by the NHIF, Sudan in 2016 were analysed. Outcome measure(s): the percentage of the cost of the ten costly medicines, the cost of the top 20% of the items.

Region covered**:** The study is a nationwide (Sudan).

Time period: 2016 - 2017

**Results:** The top ten medicines represented 24% of the total cost, while 99 medicines (out of 492 items) represented 74% of the total cost. Four out of the top ten medicines were antibiotics of which two were recommended to be used only for certain conditions and under direct supervision of the head of the unit. The cost would be reduced by one third if seventy items were purchased from local manufacturers other than to be imported. The antibiotics represented 27% of the total cost although most of them are low-priced.

**Conclusions and lessons learned:** The main strategies to reduce the cost and improve the use of medicines would be implementation of antimicrobial policy and focus on local manufacturers.

**Funding Source**

National Health Insurance Fund, Sudan

**Keywords:** Pharmaceutical, expenditure, health insurance, Sudan

**Fig. 1 (abstract P12).**
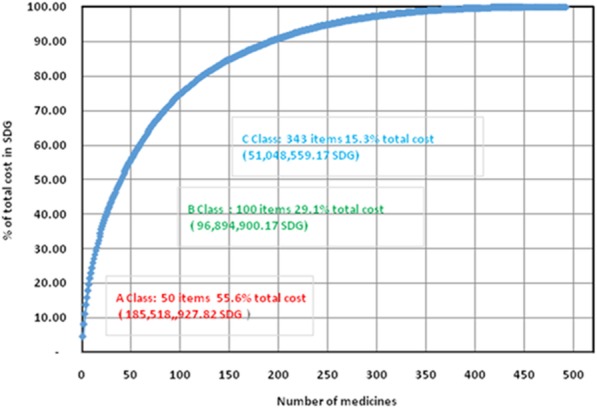
ABC analysis of usage of medicines in NHIF

**Table 1 (abstract P12).** The ten high-cost medicines in 2016

NO	Item Description	UOM	Unit price in SDG	Quantity	Total medicines cost	% of total medicines cost
1	Clopidogril 75mg tab	Tab	6.995	2,163,000.00	15,130,185.00	4.54
2	Insulin mixed	Vial	33.333	357,600.00	11,919,988.08	3.57
3	Artemether 80mg/ml injection	Amp	2.5	4,080,400.00	10,201,000.00	3.06
4	Cefiximetrihydrate 400mg capsule	Caps	3.45	2,521,880.00	8,700,486.00	2.61
5	Amoxicillin 500mg +clavulanic acid125mg. 625mg tab	Tab	1.38	5,202,104.00	7,178,903.52	2.15
6	Ceftriaxone sodium 1gm injection	Vial	8.5	718,700.00	6,108,950.00	1.83
7	Recombinant Human Erythropoietin 4000 IU/1ml for I.V, S.C	Amp	40	148,870.00	5,954,800.00	1.79
8	Amoxicillin400+ Clavulonic acid 57mg suspension (70ml/Bottle)	Bott	24	243,900.00	5,853,600.00	1.75
9	Diclofenac 75 mg inj	Amp	5	1,013,200.00	5,066,000.00	1.52
10	Artemether 40mg/ml injection	Amp	2	2,433,600.00	4,867,200.00	1.46
	TOTAL				80,981,112.60	24.28

**Fig. 2 (abstract P12).**
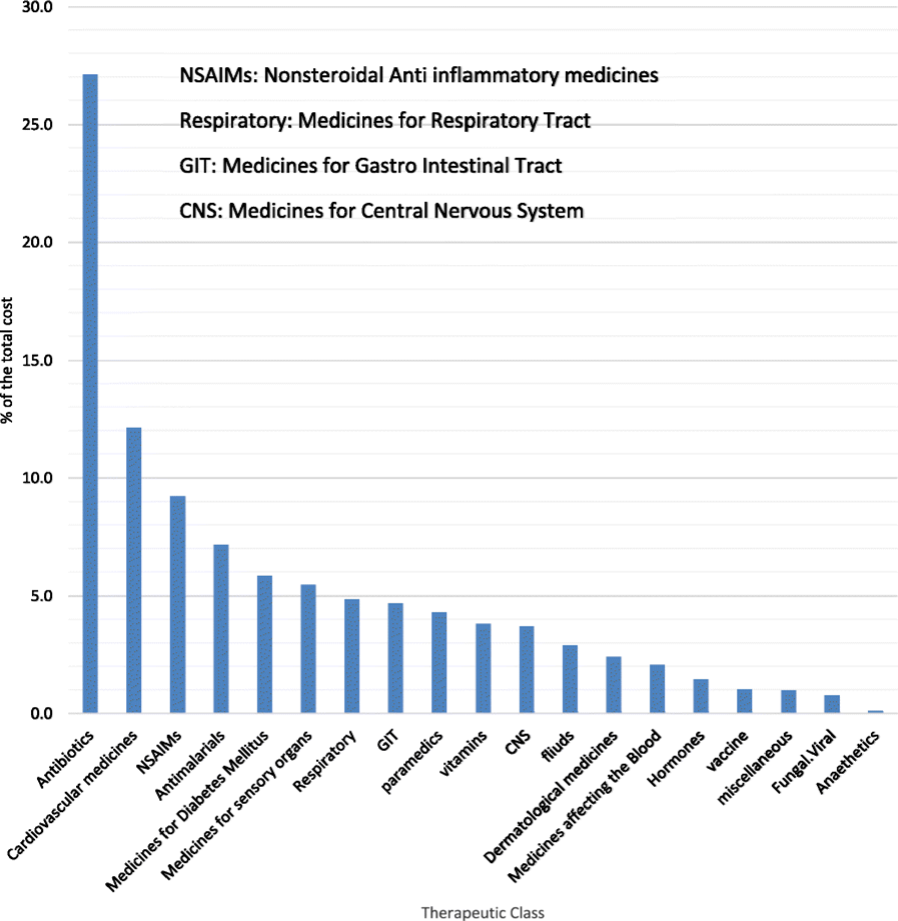
The cost of medicines by therapeutics class

### P13 Is cost-opportunity an effective strategy for drug expenditure governance? The experience on oncology drugs of the Emilia-Romagna Region, Italy

#### Lucia Magnano, Francesco Nonino, Roberta Giroldini, Elisabetta Pasi, Maria Chiara Silvani, Anna Maria Marata

##### WHO Collaborating Centre in Evidence-Based Research Synthesis and Guideline Development - Direzione Generale Cura della Persona Salute e Welfare, Servizio Assistenza Territoriale - Area Farmaco e Dispositivi Medici, Regione Emilia Romagna, Bologna, Italy

###### **Correspondence:** Francesco Nonino (francesco.nonino@regione.emilia-romagna.it)

**Background:** High cost oncology drugs challenge the sustainability of healthcare systems. In Italy, although reimbursement price is negotiated at a national level, each Italian Region implements its own drug governance policy adjusted to local needs.

**Objectives:** To describe an Italian regional governance policy on oncology drugs based on recommendations produced with the GRADE methodology [1] and monitored through quantitative indicators on expected prescription.

**Methodology:** Guidance was produced by the Gruppo Regionale Farmaci Oncologici (GReFO) multi-stakeholder oncology workgroup, committed to warranting equity, appropriate drug prescription and sustainability. For each indication, efficacy and safety of newly marketed cancer drugs are systematically appraised. Although formal cost-effectiveness analysis is not performed, cost is considered if no difference between therapeutic alternatives within the same drug class is observed. Description of the feasibility and economical impact of adding quantitative indicators of expected use and cost-opportunity issues to evidence-based recommendations on first-line treatment of advanced stage melanoma (ASM). Expected prescription figures were based on registries and extrapolated from epidemiological studies [Figure 1].

Region covered: Emilia-Romagna Region, Italy, 4-million inhabitants.

Time period: The GReFO recommendations on ASM were produced in 2017. Prescription data refer to 2018.

**Results:** In 2017, licensed monotherapies for wild-type patients with ASM were nivolumab, pembrolizumab and ipilimumab, while patients with the BRAF-V600 mutation (BRAF+) were eligible also to anti-BRAF/anti-MEK associations (BMAs). Evidence-based recommendations with the same strength and direction were issued for both nivolumab and pembrolizumab in wild-type (strong positive) and in BRAF+ (weak positive) patients. Weak positive recommendations were issued for BMAs in BRAF+ patients (Figure 1]). According to cost-opportunity issues, clinicians agreed to recommend, within the immunotherapy class, the least expensive drug in view of the substantially higher cost of pembrolizumab. In 2018, a sample of 154 ASM patients (about 70% of the total) undergoing immunotherapy, 117 (76%) were given nivolumab and 37 (24%) pembrolizumab, with a total expenditure of 5.826.509 € for both drugs. Adherence to cost-opportunity recommendation produced an estimated cost saving of about 11% (1.260.560 €) as compared with a hypothetical treatment of 50% of patients with each drug. In the case of the BMAs, the consequence of equal strength and direction recommendations led to a further discount from one of the two companies (-45%).

**Conclusions and lessons learned:** An evidence-informed drug governance policy involving multiple stakeholders and sharing context-specific issues is feasible in a public healthcare system. Cost-opportunity recommendations linked to economic incentives may result in substantial savings.

**Funding Source**

Direzione Generale Cura della Persona Salute e Welfare, Servizio Assistenza Territoriale, Area Farmaco e Dispositivi Medici, Regione Emilia Romagna, Bologna, Italy.

**Reference**

1. Atkins D, et al; GRADE Working Group. Grading quality of evidence and strength of recommendations. BMJ. 2004 Jun 19;328(7454):1490.

**Keywords:** Recommendation, GRADE method, nivolumab, pembrolizumab, melanoma

**Fig. 1 (abstract P13).**
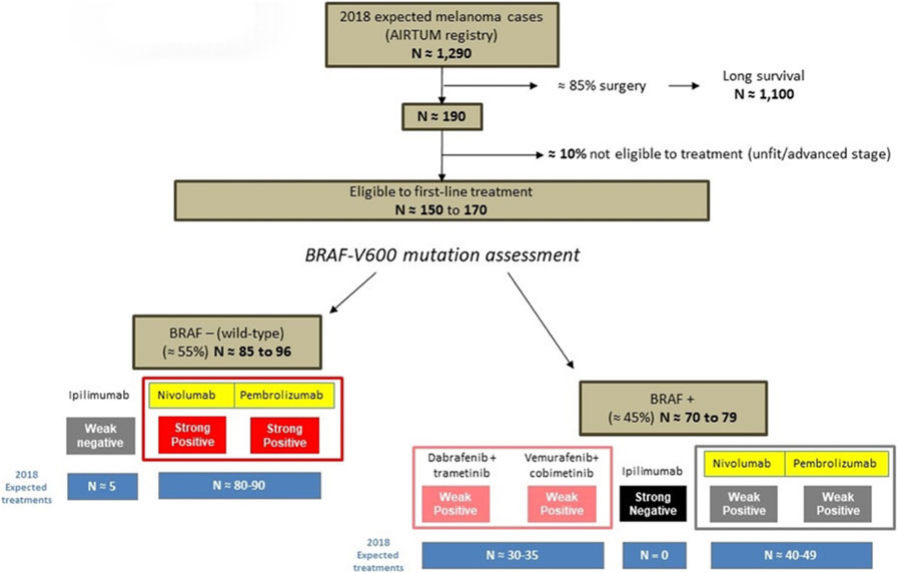
Emilia-Romagna Region decisional pathway for the first-line treatment of ASM

### P14 Assessing access to essential medicines list (EML) in the Republic of Moldova

#### Rita Seicas^1^, Ghenadie Turcanu^1^, Stela Bivol^1^, Angela Carp^2^

##### ^1^Center for Health Policies and Studies (PAS Center), Chisinau, Republic of Moldova; ²Independent consultant, Chisinau, Republic of Moldova

###### **Correspondence:** Rita Seicas (rita.seicas@pas.md)

**Background:** While Moldova has adopted policies on essential medicine list (EML), implementation has never been systematically reviewed. The PAS Center conducted a study on access to essential medicines.

**Objectives:** This study aimed to analyze implementation of the concept of essential medicines in the national health system and to improve the application into practice.

**Methodology:** The study had two components: 1) Analysis of national legislative and regulatory framework on essential medicines against international practices. 2) Analysis of alignment of the national EML (NEML) to WHO EML and the role of EML in developing the list of medicines of centralized public procurement (LMCPP). Data sources: regulatory documents for EML, updated NEML and LMCPP, bids and results of centralized medicine procurement.

Region covered: WHO EURO. The study was conducted at national level (Republic of Moldova).

Time period: 01. 01. 2018 – 28. 09. 2018

**Results:** The first NEML was approved in 1996, and was revised four times, last one in 2011. The NEML regulation was approved in 2007 and is in need of significant revisions to: (1) improve comprehensive implementation EML; (2) establish an explicit algorithm for inclusion and exclusion of molecules in NEML; (3) develop separate NEML for adults and children; (4) structure NEML in chapters based on levels of healthcare services; (5) establish a mechanism for participation of the healthcare and pharmaceutical community, patient associations and consumers in the process of NEML development and review; (6) set a monitoring and evaluation (M&E) framework for EML implementation. The analysis of the NEML in 1996 versus 2011 reveals that the number of medicines has expanded considerably during this time (Table 1). Comparative analysis of the NEML (635 molecules) with 2017 WHO EML reveals that 337 molecules are common to both lists, 152 molecules of WHO EML missing in 2011 NEML and 263 molecules of NEML not part of WHO EML. The LMCPP contains 52% of international non-proprietary names (INNs) from EML (289 INNs out of a total of 560 procured INNs) in 2017, representing an improvement compared to 41% in 2011. The share of public budget for procurement of EML in the total contracted amount for public procurement of medicines has increased: from 43% in 2011 to 65.9% in 2017.

**Conclusions and lessons learned:**

The NEML in Moldova is outdated. Public procurements show a low share of NEML-listed medicines out of the LMCPP. This is a lost opportunity to ensure access and value for money and compliance with the WHO EML.

**References:**

1. MoH Order no. 162 of 23.04.2017 regarding the approval of the Regulation and the List of Essential Medicines.

2. WHO EML, 20^th^ edition, 2017

3. National Tenders on Public procurement of medicines

**Keywords:** Essential medicines list, public procurement, pharmaceutical regulation, drug policy

**Table 1 (abstract P14).** National Essential Medicines List (NEML) evolution 1996-2011

Year of NEML approval	Total number of molecules (excluding duplicates)	Total number of molecules (including duplicates)	Total number of pharmaceutical forms	Rate of pharmaceutical form per molecule	Total number of therapeutic categories
1996	106	108	147	1.36	0
2007	475	504	718	1.42	29
2009	519	578	819	1.41	27
2011	576	635	856	1.34	29

### P15 Regulating medicine prices in Morocco - Effects of the medicine price decree 2014 on medicine prices

#### Bouchra Benslaoui^1^, Mohammed Wadie Zerhouni^1^, Anas Chikhaoui^1^, Fatima Zahra Ben Fouila^2^, Katharina Habimana^3^, Hafid Hachri^4^, Maryam Bigdeli^4^, Sabine Vogler^3^, Jamal Taoufik^1^, Hicham Nejmi^2^

##### ^1^Medicine and Pharmacy Directorate, Ministry of Health, Rabat, Morocco; ²General Secretary, Ministry of Health, Rabat, Morocco; ³WHO Collaborating Centre for Pharmaceutical Pricing and Reimbursement Policies, Gesundheit Österreich GmbH (GÖG / Austrian Public Health Institute), Vienna, Austria; ^4^WHO Country Office Morocco, Rabat, Morocco

###### **Correspondence:** Katharina Habimana (katharina.habimana@goeg.at)

**Background:** In December 2013, the Moroccan government reviewed the medicine price regulation. In April 2014, the Ministry of Health implemented a decree for new provisions related to external price referencing, a generic price link and changes in wholesale and pharmacy margins affecting the conditions and modalities for setting the pharmacy retail price of locally manufactured and imported medicines.

**Objectives:** The objective of this price data study is to evaluate the effects of the revision of the medicine price regulation implemented in Morocco in 2014 on the different levels in the pharmaceutical supply chain (manufacturers, wholesalers, pharmacies) as well as on the health insurance agencies and on the accessibility and affordability of medicines for citizens.

**Methodology:** A data set consisting of more than 7,000 medicines (all medicines on the Moroccan market), that contains medicine price data for different price types (public prices; pharmacy retail prices; pharmacy purchasing prices = wholesale prices; ex-factory prices) and further characteristics (e.g. therapeutic class, originator/generic, reimbursed/non-reimbursed) was analysed with a view to measuring the medicine price situation before and after the implementation of the decree. In particular, the investigation aimed to disentangle the effects of the new decree on various actors (manufacturers, wholesalers, pharmacies) and on the accessibility to medicines for citizens. The analysis is descriptive.

Region covered: Morocco

Time period: Before April 2014 – April 2019

**Results:** Preliminary results for the overall data set show that, on average, the differences between prices before the decree and current prices varied between 7% and 11% depending on the price type. On average, wholesalers and manufacturers were confronted with higher degrees of price reduction in percentage of their prices, whereas, on average, pharmacists did not as much reduce their prices for the citizens. Yet, pharmacists had higher price reductions as percentage of their pharmacy prices for high priced medicines with lower volume, while manufacturers observed higher price reductions for lower priced medicines with higher volume.

**Conclusions and lessons learned:** Pharmacy purchasing prices showed, on average, highest reduction (approx. 11%), while the decreases were lowest at pharmacy retail price level (approx. 7%). Possible explanations are yet to be explored. A slightly lower reduction, on average, in ex-factory prices compared to the decrease in pharmacy purchasing prices points to possibly higher cuts in wholesale margins. These are preliminary findings that will be refined (results available in October 2019), that will show further, more detailed results with regard to different categories of medicines (reimbursable vs. non-reimbursable, originator vs. generic, imported vs. fabricated, medicines per therapy etc.).

**Funding Source**

WHO Country Office for Morocco

**Keywords:** Morocco, medicine prices, evaluation

## POSTER PRESENTATIONS STRAND 2

### P16 How the Euripid Collaboration contributes to the affordability of medicines in Europe

#### Claudia Habl^1^, Gergely Németh^2^, Peter Schneider^1^

##### ^1^Gesundheit Österreich GmbH (GÖG / Austrian Public Health Institute), Vienna, Austria; ²Hungarian National Health Insurance Fund - NEAK, Budapest, Hungary

###### **Correspondence:** Claudia Habl (claudia.habl@goeg.at)

**Background:** EURIPID (www.euripid.eu) is a voluntary collaboration between European countries to run a database with information on national prices of pharmaceuticals in a standardised format. It makes prices of publicly funded medicines more transparent via an online accessible and reliable 24-hour-database.

**Objectives:** The comparison of the prices of pharmaceuticals is an important element of a popular policy in Europe, called ‘External Reference Pricing’ (ERP). Albeit being a commonly used policy instrument, ERP is often challenged by stakeholders by claiming potential negative effects on patients’ access or that it is hampering uptake of medicinal products in a market. The Euripid Collaboration developed, triggered by a call of the European Parliament to include real prices in Euripid (1), recommendations which are meant to guide a coordinated approach of national competent authorities regarding the use of ERP to mitigate potential negative impact for patient access.

**Methodology:** A study team led by the Austrian Public Health Institute (GÖG) and the Hungarian National Health Insurance Fund (NEAK) developed a Technical Guidance Document on ERP of medicines.

The Guidance Document (2) was, based on a scientific literature analysis and a collection of best practices (3), prepared following a series of formal and informal consultations including Face-to-Face workshops between Euripid members, the PPRI network, EC-representatives and stakeholders in the field, e.g., economic operators. The Euripid Collaboration formally endorsed the use of the principles within the remit of their responsibilities in summer 2018.

**Region covered**: EEA and Israel.

**Time period**: 2016-2018

**Results:** The 12 principles state, e.g., that a) ERP is an important policy tool that should be used in a mix with other instruments and not as stand-alone policy tool, b) that ERP should take place on a single product basis rather than by indices and that c) evidence has shown that ERP is most effective when applied to pharmaceuticals without generic or therapeutic competition and d) that ERP procedures should be performed with the highest possible accuracy and completeness of data sources.

**Conclusions and lessons learned:** The principles are an important step towards a more balanced use of ERP policy and thus a higher acceptance in Europe. The Euripid database is aiding countries to perform price comparisons for ERP or price monitoring in a standardised format. One recommendation referred to a continuation of information exchange in the area of pricing of medicines between the Euripid collaboration and stakeholders which led to the establishment of a stakeholder dialogue platform in 2019. Efforts were also made to improve affordability of medicines by better transparency of prices of medicines as contained in Euripid. This aspect is especially interesting in the light of the recent WHO resolution (4) that urges countries to take appropriate measures to publicly share information on the net prices of health products.

**Funding Source**

Euripid Collaboration (26 countries, European Commission)

**References**

1) Report of the European Parliament on EU options for improving access to medicines 2016 - http://www.europarl.europa.eu/doceo/document/A-8-2017-0040_EN.html?redirect

2) Euripid Guidance Document 2018 - https://ppri.goeg.at/sites/ppri.goeg.at/files/inline-files/EURIPID_GuidanceDocument_V8.1_310718_5_0.pdf

3) Euripid Best Practice Report 2017 - https://ppri.goeg.at/sites/ppri.goeg.at/files/inline-files/Euripid_BestPracticeReport_final_May2017_2.pdf

4) WHO Resolution 2019 - https://www.healthpolicy-watch.org/wp-content/uploads/2019/04/WHA-Resolution_DRAFT_29. 4.2019.pdf

**Keywords:** ERP, governance, medicinal products, medicines prices, affordability

### P17 Estimating price developments of biological medicines during market exclusivity

#### Peter Schneider^1^, Lena Lepuschütz^2^, Nina Zimmermann^1^, Sabine Vogler^1^

##### ^1^WHO Collaborating Centre for Pharmaceutical Pricing and Reimbursement Policies, Pharmacoeconomics Department, Gesundheit Österreich GmbH (GÖG / Austrian Public Health Institute), Vienna, Austria; ²Health Economics and Health Systems Analysis, Gesundheit Österreich GmbH (GÖG / Austrian Public Health Institute), Vienna, Austria

###### **Correspondence:** Peter Schneider (peter.schneider@goeg.at)

**Background:** A medicine passes through several different stages which is known as a ‘product life cycle’. Each stage is embedded in a regulatory and policy environment, which determines price dynamics. While there is abundant literature on prices of chemical medicines in the off-patent sector, analyses on prices for biological medicines is scarce.

**Objectives:** The aim of the study was to estimate price developments of biological medicines during the stage of market exclusivity and compare these results with list prices of biologicals prior to the entry of the first biosimilar.

**Methodology:** The estimation of pharmaceutical price developments was based on a discrete-event simulation (DES) which used information on the EPR mechanisms and characteristics of the included countries (28 EU Member States plus Switzerland and Norway). The model ran over a 10 year time horizon which is the minimum number of years of market protection in the EU. The Pharmaceutical Price Information (PPI) service provided list prices of two biological medicines (Adalimumab and Rituximab) in the months before the first biosimilar entered the market.

Region covered: 28 EU Member States plus Switzerland and Norway.

Time period: Prices were analysed at the month prior to launch of the first Biosimilar in one European market, i.e. January 2018 for Adalimumab and November 2016 for Rituximab.

**Results:** The model estimated that the average price level fell consecutively over the years as more and more countries had received and re-evaluated a price based on the average, minimum or other arithmetic measure of existing prices. After ten years, the average price level over the 30 countries was 80.2% of the starting price. The highest price countries - excluding countries for which prices were assumed to be fixed - were Austria, Belgium, Luxembourg and Switzerland, while lower prices were predicted in Spain, Romania and Croatia. In comparison to the model’s estimations, the average price level of list prices was 66.8%. The countries with the highest price level were Germany, Switzerland and Poland, while lowest prices were observed in UK, France and Greece.

**Conclusions and lessons learned:** The model made several simplifying assumptions which had a large impact on the estimated price level of medicines over the product life cycle and partly explain observed differences to list prices.

**Funding Source**

Health Programme of the European Union; Federal Ministry of Labour, Social Affairs, Health and Consumer

**Keywords:** European price comparison, biosimilars, off-patent price developments

### P18 Choosing the right medicines for price comparisons - Analysis of prices of pharmaceutical presentations of the same active ingredient

#### Sabine Vogler, Peter Schneider

##### WHO Collaborating Centre for Pharmaceutical Pricing and Reimbursement Policies, Pharmacoeconomics Department, Gesundheit Österreich GmbH (GÖG / Austrian Public Health Institute), Vienna, Austria

###### **Correspondence:** Sabine Vogler (sabine.vogler@goeg.at)

**Objectives:** The study aimed to analyse the prices of different pharmaceutical presentations of the same active ingredient in European countries with a view to assessing the possible differences between them.

**Background:** The selection of medicines is a key methodological decision of any international price comparison. In particular, it has been discussed whether, or not, given widespread use of flat pricing, a single pharmaceutical presentation is sufficient to represent the active ingredient, or if all presentations of an active ingredient should be included in a study.

**Methodology:** Prices of originator medicines of 22 active ingredients were surveyed and analysed in 27 European countries (all European Union Member States except Malta). For all active ingredients, at least two presentations (e.g. with difference in strengths, pharmaceutical form or primary/immediate packaging such as pen and syringe) were studied. At least one presentation of the selected active ingredients ranked among the high-cost medicines for Austrian public payers in Q2/2017. Medicine price data were collected through the Pharma Price Information (PPI) service of the Austrian Public Health Institute and were surveyed as of September 2017. Data analysis was done for ex-factory prices (list prices, before any discounts) per unit (e.g. per tablet, vial) to account for differences in the pack size.

Region covered: 27 European Union Member States (WHO European region)

Time period: Price data were surveyed as of September 2017.

**Results:** For 18 of the 22 studied active ingredients, the per unit ex-factory prices were the same for the surveyed pairs of the pharmaceutical presentations in several countries. As a result, the relative ranking of unit prices across the European countries did not differ considerably between presentations of the same active ingredient (see Figure 1). A different pattern was found in cases of the marketing of different presentations for different indications (denosumab) and of emerging generic competition, which also impacted originator prices (rosuvastatin).

**Conclusions and lessons learned:** The findings suggest that for medicines in the on-patent market the inclusion of a single presentation per active ingredient in a price comparison can be sufficient, since prices do not substantially differ. As soon as generic competition starts, however, price dynamics will likely occur, and it is recommended to include further pharmaceutical presentations of an active ingredient in a medicine price study.

**Funding Source**

This is a follow-up analysis of a European medicine price study performed for the Austrian Federal Ministry of Labour, Social Affairs, Health and Consumer Protection. The Pharma Price Information (PPI) service, from which medicine price data were sourced, is also financially supported by the Austrian Federal Ministry of Labour, Social Affairs, Health and Consumer Protection.

**Keywords:** Medicine price, methodology, price study, cross-country comparison, product selection

**Fig. 1 (abstract P18).**
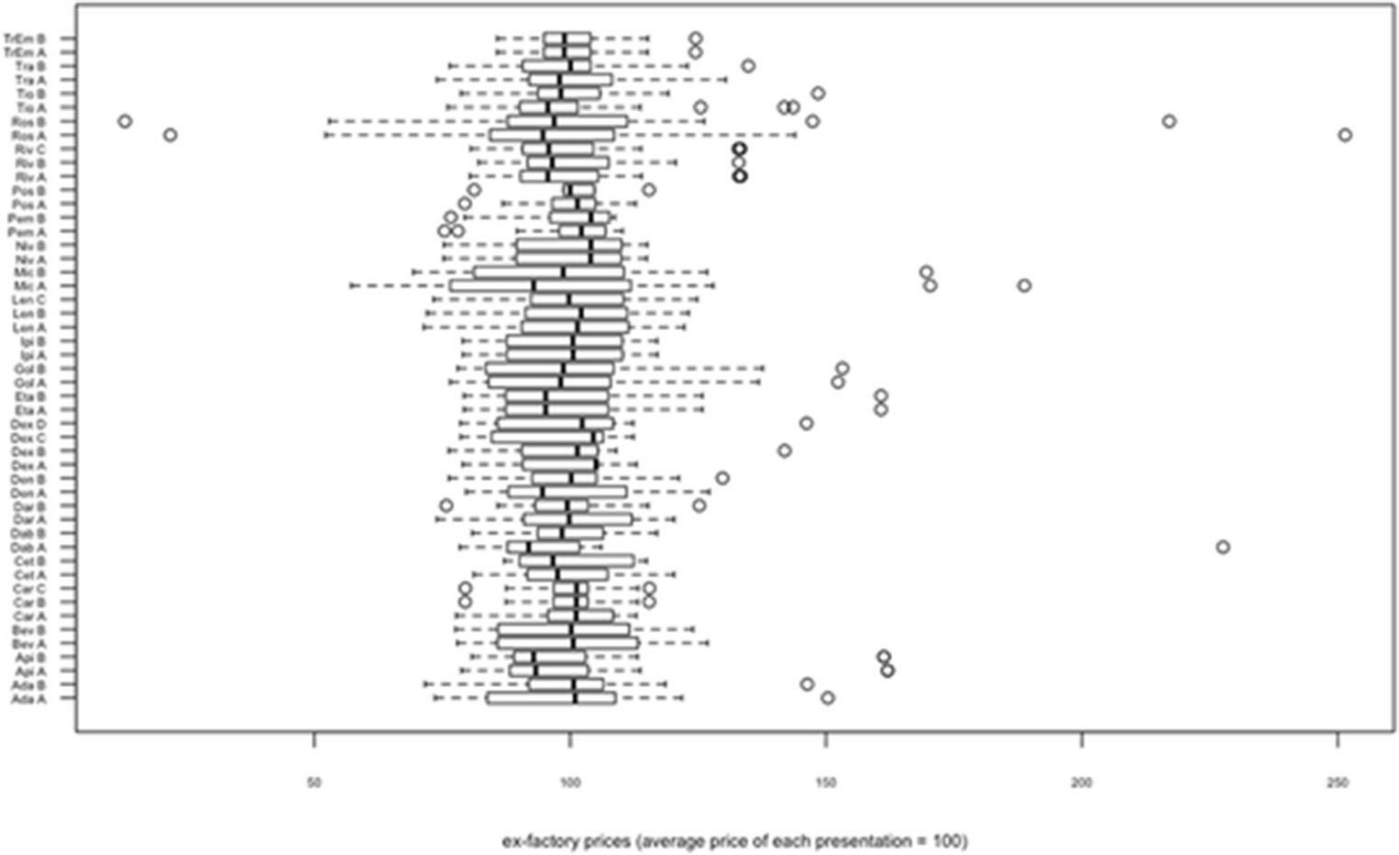
Ex-factory prices of at least 2 pharmaceutical presentations of the same active ingredient for 27 EU Member States, 2017 (average price of each presentation = 100). **Notes:** The average price of each presentation defined as an index (= 100). The box corresponds to the area in which the middle 50% of the data are located (interquartile distance). The black line describes the location of the median. The dashed whiskers are limited to 1.5 times the length of the interquartile range. The circles stand for statistical outliers. The analysis was run for all 27 European Union Member States (all but Malta). Included presentations: Ada = adalimumab 40 mg, 0.8 ml, injection for solution, 2 pre-filled syringes (A), adalimumab 40 mg, 0.8 ml, injection for solution, 2 pre-filled pens (B); Api = apixaban 2.5 mg, 60 f/c tablets (A), apixaban 5 mg, 60 f/c tablets (B); Bev = bevacizumab 100 mg / 4 ml concentrate to produce a solution for infusion, 1 vial (A), bevacizumab 400 mg / 16 ml concentrate to produce a solution for infusion, 1 vial (B); Car = carfilzomib 60 mg / 30 ml powder for solution for infusion, 1 vial (A), carfilzomib 10 mg / 5 ml powder for solution for infusion, 1 vial (B), carfilzomib 30 mg / 15 ml powder for solution for infusion, 1 vial (C); Cet = cetuximab 100 mg / 20 ml solution for infusion, 1 vial (A), cetuximab 500 mg / 100 ml solution for infusion, 1 vial (B); Dab = dabigatran etexilate 110 mg, 30 hard capsules (A), dabigatran etexilate 150 mg, 30 hard capsules (B), Dar = daratumumab 100mg / 5 ml concentrate to produce a solution for infusion, 1 vial (A), daratumumab 400mg / 20 ml concentrate to produce a solution for infusion, 1 vial (B); Den = denosumab 60 mg / 1 ml solution for injection, 1 pre-filled syringe (A), denosumab 120 mg / 1.7 ml solution for injection, 1 vial (B); Dex = dexmedetomidine 200 mcg / 2 ml concentrate to produce a solution for infusion, 5 ampoules (A), dexmedetomidine 200 mcg / 2 ml concentrate to produce a solution for infusion, 25 ampoules (B); Eta = etanercept 50 mg / 1 ml solution for injection, 4 pre-filled syringes (A), etanercept 50 mg / 1 ml solution for injection, 4 pre-filled syringes (B); Gol = golimumab 50 mg / 0.5 ml solution for injection, 1 pre-filled pen (A), golimumab 50 mg / 0.5 ml solution for injection, 1 pre-filled syringe (B); Ipi = ipilimumab 50 mg / 10ml concentrate to produce a solution for infusion, 1 vial (A), ipilimumab 200 mg / 40 ml concentrate to produce a solution for infusion, 1 vial (B); Len = lenalidomid 10 mg, 21 hard capsules (A), lenalidomid 15 mg, 21 hard capsules (B); Mic = micafungin 50 mg / 10 ml powder for a concentrate to produce a solution for infusion, 1 vial (A), micafungin 100 mg / 10 ml powder for a concentrate to produce a solution for infusion, 1 vial (B); Niv = nivolumab 40 mg / 4 ml concentrate to produce a solution for infusion, 1 vial (A), nivolumab 100 mg / 10 ml concentrate to produce a solution for infusion, 1 vial (B); Pem = pembrolizumab 50 mg / 2 ml powder for a concentrate to produce a solution for infusion, 1 vial (A), pembrolizumab 100 mg / 4 ml powder for a concentrate to produce a solution for infusion, 1 vial (B); Pos = posaconazole 100 mg, 24 enteric tablets (A), posaconazole 100 mg, 96 enteric tablets (B), Riv = rivaroxaban 20 mg, 30 film-coated tablets (A), rivaroxaban 20 mg, 30 film-coated tablets (B); Ros = rosuvastatin 10 mg, 30 film-coated tablets (A), rosuvastatin 20 mg, 30 film-coated tablets (B); Tio = tiotropium bromid 18 mcg inhalation powder, 30 capsules (A), tiotropium bromid 2.5 mcg inhalation solution, 1 inhaler (B); Tra = trastuzumab 150 mg / 7.2 ml powder for a concentrate to produce a solution for infusion, 1 vial (A), trastuzumab 120 mg / 5 ml a solution for injection, 1 vial (B); TrEm = trastuzumab emtansine 100 mg / 5 ml powder for a concentrate to produce a solution for infusion, 1 vial (A), trastuzumab emtansine 160 mg / 8 ml powder for a concentrate to produce a solution for infusion, 1 vial (B)

## POSTER PRESENTATIONS STRAND 3

### P19 Innovative policy options to secure access to medicines – a literature review

#### Nora Franzen, Valesca P Retèl, Winnie Schats, Wim H van Harten

##### The Netherlands Cancer Institute, Amsterdam, The Netherlands

###### **Correspondence:** Nora Franzen (n.franzen@nki.nl)

**Background:** Access to medicines is a core component of the right to health. High expenditure on innovative cancer drugs threatens this right and, considering finite resources, the financial sustainability of cancer care. Innovative solutions are therefore needed and highly discussed. However, despite the scientific and public interest, publications are often opinion-based.

**Objectives:** The objective of this study was to facilitate an evidence-based discourse on innovative policy options to reduce drug prices at market launch. We reviewed the literature to produce an inventory of policy options and analyzed their quantitative evidence to select promising solutions.

**Methodology:** We performed a scoping review, selecting for studies that propose solutions with either a direct or obvious indirect impact on pharmaceutical prices at market launch, with relevance to oncology and high-income countries. We created an inventory of policy options, categorized publications according to their evidence-base and analyzed quantitative articles. We selected promising options and collected feedback from a survey among European experts in the field of oncology and health regulation

Region covered: We screened globally and selected for the EURO region. The scope of many policy options is however global.

Time period: 2017-2019

**Results:** We screened 4775 articles and selected 85 articles that we used to produce an inventory of policy options in the intellectual property, pricing, and the research & development environment. 22 articles used a quantitative approach but the overall evidence level per policy was low. Based on system disruption, market mechanisms, and potential price impact, we identified seven promising solutions of which experts prioritized transparency and combined purchasing. Two-part-pricing and de-linkage were the most controversial proposals.

**Conclusions and lessons learned:** Despite the importance of finding solutions that secure access to medicines, a coordinated approach to structurally evaluate proposals is lacking. Quantitative methods are rarely used, and current evidence is insufficient to structurally evaluate proposals. We advise testing proposals with small-scale experiments, dynamic simulations, and policy pilots.

**Keywords:** Drug prices, innovative policy proposals, evidence-based policy making

### P20 Integrating public preferences into reimbursement decisions: case studies from Belgium and New Zealand

#### Christine Leopold, Christine Y. Lu, Anita K. Wagner

##### Department of Population Medicine, Harvard Medical School and Harvard Pilgrim Health Care Institute Boston, MA, USA

###### **Correspondence:** Christine Leopold (christleopold@gmx.net)

**Background:** Public health care payer organizations face increasing pressures to make transparent and sustainable coverage decisions about ever more expensive prescription drugs, suggesting a need for public engagement in coverage decisions. However, little is known about countries’ approaches to integrating public preferences in existing funding decisions.

**Objectives:** The aim of this study is to explore how Belgium and New Zealand used deliberative processes to engage the public to change their public reimbursement system and to identify lessons learned from these countries’ approaches.

**Methodology:** We used a qualitative study design to describe these two countries’ deliberative processes. We first reviewed key country policy documents and then conducted semi-structured interviews with in total five senior system leaders from Belgium and New Zealand. We assessed each country’s rationales for and approaches to engaging the public in pharmaceutical coverage decisions and identified lessons learned. We used qualitative content analysis of the interviews to describe key themes and subtopics.

**Region covered:** This study includes an assessment of national policies of Belgium (EURO) and New Zealand (WPRO).

Time period: January 2017 - June 2017

**Results:** In both countries, the national public payer organization initiated and led the process of integrating public preferences into national coverage decision-making. Reimbursement criteria considered outdates and changing societal expectations prompted the change. Both countries chose a deliberative process of public engagement with a multi-year commitment of many stakeholders to developing new reimbursement processes. Both countries’ new reimbursement processes put a stronger emphasis on quality of life, the separation of individual versus societal perspectives, and the importance of final reimbursement decisions being taken in context rather than based largely on cost-effectiveness thresholds.

**Conclusions and lessons learned:** To face the growing financial pressure of sustainable funding of medicines, Belgium’s and New Zealand’s public payers have developed processes to engage the public to define what a reimbursement system's priorities are. While these countries differ in context and geographic location, they still came up with overlapping lessons learnt which include the need for 1) political commitment to initiate change, 2) broad involvement of all stakeholders and 3) commitment of all to engage in a long-term process. To evaluate these changes, further research is required to understand how coverage decisions in systems with and without public engagement differ.

**Funding Source**

Austrian Science Fund (FWF): project number: J-3684

**Keywords:** Public preferences, insurance coverage, prescription drugs, health care, qualitative research

### P21 Ex-post analysis of medicines subject to Managed-Entry-Agreements (MEAs) – a feasible approach for monitoring and price analyses

#### Peter Schneider^14^, Claudia Habl^24^, Nemeth Gergely^34^

##### ^1^WHO Collaborating Centre for Pharmaceutical Pricing and Reimbursement Policies, Pharmacoeconomics Department, Gesundheit Österreich GmbH (GÖG / Austrian Public Health Institute), Vienna, Austria; ²Gesundheit Österreich GmbH (GÖG / Austrian Public Health Institute), Vienna, Austria; ³National Institute of Health Insurance Fund Management (Nemzeti Egészségbiztosítási Alapkezelő, NEAK), Budapest, Hungary; ^4^Executive Committee of the EURIPID Collaboration, Budapest, Hungary

###### **Correspondence:** Peter Schneider (peter.schneider@goeg.at)

**Background:** Research in the field of medicine prices requires decisions on the methods applied in the study. Five major – but not exclusive – dimensions of methodological designs are (1) geographic area, (2) sector/setting, (3) range of products, (4) price type, and (5) timing of the price data. The decisions on certain approaches are often determined by the study purpose, objectives and perspective, but the main goal is to make meaningful comparisons.

**Objectives:** The aim of the survey was to assess which information competent authorities, researchers and stakeholder in the field of pharmaceutical pricing need when they conduct price analyses.

**Methodology:** A needs assessment survey has been conducted among competent authorities and stakeholders in the field of pharmaceutical policy. The questionnaires contained 30 items and was structured in five overall topics. These topics were (1) General Information about the respondent (2) Type of products and type of prices subject to price comparison, (3) Procedures of price comparison, (4) Methodological issues of price comparisons, and (5) further relevant information the respondent wanted to share. The questionnaire was distributed to 90 persons from 56 national and European institutions and associations.

Region covered: Europe, EU28

Time period: March 2016 - May 2016

**Results:** The survey was completed by 24 institutions, of which 15 were competent authorities for pricing and reimbursement. The other nine answers were provided by international organisations, European associations of affected stakeholders and experts on pricing and reimbursement. Respondents emphasised the importance of making meaningful comparisons of medicine prices, and highlighted that more information may support this goal. 16 respondents identified information about the existence of Managed Entry Agreements (MEAs) as a supportive piece of information for meaningful comparisons, and if this is not possible, respondents would like to know if (statutory) discounts, rebates and claw-backs are applied.

**Conclusions and lessons learned:** In the last couple of years actors in the field of pharmaceutical pricing (competent authorities, stakeholders and researchers) have established methods to conduct price analyses. Despite varying approaches in those methods, the unifying assumption was the information value of list prices. The increasing practice of MEAs, has shattered the backbone of price comparison into pieces and - due to believed benefits of MEA - it will not change in the near future. As legal requirements obstruct any meaningful comparisons for medicines subject to MEAs, competent authorities should consider ways to enable ex-post analysis of effective prices when those contracts terminate i.e. realignment of list prices to effective prices.

**Funding Source**

Health Programme (2014 - 2020) of the European Union

**Keywords:** Price comparisons, price analyses, Managed Entry Agreements (MEA)

### P22 When Less Means More: Insight into the Spending on Expensive Drugs for Rare Diseases

#### Tanya Potashnik, Elena Lungu

##### Patented Medicine Prices Review Board, Policy Development, Ottawa, Canada

###### **Correspondence:** Tanya Potashnik (tanya.potashnik@pmprb-cepmb.gc.ca)

**Background:** An increasing number of drugs for rare diseases have emerged in recent years, bringing hope to patients suffering from life-threatening or debilitating conditions. However, most come with price tags that patients cannot afford and payers struggle to fund. With few or no therapeutic comparators and uniformly high prices internationally, the recent trends in expensive drugs for rare diseases (EDRDs) pose important challenges around affordability, access, and long-term system sustainability.

**Objectives:** This analysis aimed to identify the major factors driving EDRD spending and their mounting importance in Canadian and OECD markets.

**Methodology:** Using sales data from IQVIA’s MIDAS™ Database, this analysis provides insight into the EDRD market, with information on availability, pricing, sales uptake, and market shares in Canada and internationally. The results touch on the relationship between treatment cost and the size of the treatment population, assessing these aspects against past trends. The analysis also includes an overview of health technology assessments, the status of the pan-Canadian Pharmaceutical Alliance (pCPA) price negotiations, and Canadian public drug plan reimbursement for EDRDs.

Region covered: International markets examined include the countries in the Organisation for Economic Co-operation and Development (OECD), highlighting Canada and its comparator markets.

Time period**:** The analysis focused on 2018, with retrospective trends dating back two decades.

**Results:** This study analyzed 79 EDRDs, split almost equally into oncology and non-oncology medicines. The treatment costs for most non-oncology EDRDs exceeds a staggering $300,000 annually, while most EDRDs for oncology exceed $11,000 per 28-day course. Preliminary data suggests that despite the small patient populations they treat, EDRDs are a $1.8 billion market in Canada, owing to their remarkably high prices. With sustained annual rates of increase of over 30%, this is a fast-growing market bearing a constant inflow of specialty products. In 2016 and 2017 alone, around two dozen oncology and non-oncology EDRDs were approved in Canada, and the profile of the pipeline supports a perpetuation of these trends. These drugs, along with future launches, are expected to be a significant driver of pharmaceutical spending.

**Conclusions and lessons learned:** Given the high price and increased availability of EDRDs, and their importance to patients facing severe and often life-threatening diseases, this is a therapeutic area that requires a fix for the future through innovative approaches to the policy and greater international collaboration and alignment.

**Funding Source**

Government of Canada

**Keywords:** EDRDs, orphan drugs, high-cost drugs, policy, Canada

### P23 The Patented Medicine Prices Review Board Guidelines Modernization

#### Tanya Potashnik, Elena Lungu

##### Patented Medicine Prices Review Board, Policy Development, Ottawa, Canada

###### **Correspondence:** Tanya Potashnik (tanya.potashnik@pmprb-cepmb.gc.ca)

**Background:** In the last twenty years, the global environment for pharmaceuticals has shifted significantly toward emerging higher cost drugs, such as biologics and gene therapies, which have put mounting pressure on drug spending. This pressure is exacerbated by the growing discrepancy between prices listed publicly and those actually marketed resulting from an increasing use of confidential discounts and rebates. In this environment, Canadians find themselves paying some of the highest drug prices in the world, behind only the United States and Switzerland, and lacking negotiating power for drugs that have few or no therapeutic options.

**Objectives:** To discuss the policy intent of the amendments to the Canadian Patented Medicines Regulations and the adoption of a risk-based approach to regulating drug prices.

**Methodology:** The Canadian government has made improving the affordability of medicines one of its top priorities, and is enhancing its regulatory price regime. These enhancements include: (i) protecting consumers by benchmarking domestic prices against countries with similar consumer protection priorities, economic wealth and marketed medicines as Canada; (ii) regulating actual drug prices being paid in Canada and not just the non-transparent manufacturer list prices; and (iii) considering the value and the affordability of a medicine when setting the maximum price.

Region covered: The analysis focuses on Canada, with some supporting analysis of selected OECD countries.

Time period**:** The discussion focuses on the new regulations and the modernization of the guidelines with the expected implementation targeted starting with July 2020.

**Results:** Under the regulatory changes, the PMPRB will continue to establish price ceilings based on internal and external price referencing, but with an updated list of comparator countries. In addition, the PMPRB will employ a risk-based approach to price regulation, exercising further scrutiny for medicines with the greatest market power and potential for charging an excessive price. For these medicines, the PMPRB will establish a confidential rebated price ceiling derived based on pharmacoeconomic and affordability considerations.

**Conclusions and lessons learned:** This discussion will highlight key challenges for Canada, elements of the regulatory changes, and how the PMPRB envisions their implementation. By rethinking its price regulatory framework, the PMPRB seeks to contribute to a sustainable pharmaceutical system and greater access to medicines through improved affordability.

**Funding Source**

Government of Canada

**Keywords:** Policy, price regulation, high cost drugs, access, Canada

### P24 An Examination and Assessment of the Processes Involved in Setting Reimbursement Prices for Medicines in Ireland

#### Declan Bradley

##### Health Service Executive (HSE), Dublin, Ireland

###### **Correspondence:** Declan Bradley (declan.bradley@hse.ie)

**Background:** The Irish Health Service faces significant future challenges with growing costs of new medicines, combined with a pipeline of highly expensive medicines. Non-transparent commercial arrangements have helped manage the adoption and funding of expensive medicines. The 2016 IPHA Framework Agreement, was anticipated to achieve significant savings, in part through Schedule 5 which ensures that list prices of all medicines will be realigned, downwards only.

**Objectives:** This study examined processes involved in setting reimbursement prices for new medicines whilst determining the financial benefits from having an assessment and commercial negotiation process. The extent to which price realignments over time improved transparency of commercial arrangements and the long-term commercial impact of commercial negotiations at application stage were assessed. This study sought to assess whether or not there are more appropriate or efficient means of setting reimbursement prices for medicines in Ireland, determining if and what the financial benefits of the overall processes are and addressing the benefit of offsetting the transparency of pricing in favour of achieving savings.

Region covered: This study is from the perspective of the Corporate Pharmaceutical Unit (CPU) in the Primary Care Reimbursement Service (PCRS) of the Health Services Executive (HSE) in Ireland.

**Results:** From a sample of 25 commercially confidential agreements, commercial discounts ranged between 5% and 60%. Agreements consisted of budget caps, discounts off list prices and tiered discounts. Most agreements included discounts off the list price collected through rebates. Forecasts estimated commercial agreements to last from less than 500 days to almost 3500 days. The majority (72%) of medicines realigned downward in price annually, in 2016, 2017 and 2018. 12% (n=3) of medicines have realigned below their non-transparent commercially agreed price. The average time taken to reimbursement decreased year on year. Up to November 2018, a sum of almost €50 million had been collected from rebates due to commercial arrangements.

**Conclusions and lessons learned:** Annual realignments and commercial arrangements have proven beneficial to the Irish State with significant savings made. CPU has played an integral role in negotiating confidential agreements with pharmaceutical companies. Transparent pricing would be preferable but is challenging given international reference pricing constraints. The process for setting reimbursement prices in Ireland is robust and this study goes some way to support that. Nevertheless, with significantly greater challenges expected in future, additional measures are required.

**Keywords:** Commercial arrangements, confidential, realignments, transparency

**Funding Source**

None

### P25 Joint Procurement- Learnings from a pilot of joint procurement of older products

#### Helle Pasgaard Rommelhoff, Lars Ole Madsen, Dorthe Bartels, Lise Grove, Trine Ann Behnk

##### Amgros I/S, Kopenhagen, Denmark

###### **Correspondence:** Trine Ann Behnk (tab@amgros.dk)

**Background:** Denmark decided to take part in a joint procurement pilot with Norway and Iceland to seek solutions for some of the supply issues in Denmark. This was a consequence of being a small volume market with potentially limited attractivity for suppliers of older products. An initial evaluation of synergies and discrepancies among the involved countries supported the understanding of how to jointly procure medicines for the hospital sector.

**Objectives:** To share learnings from a pilot of joint procurement across borders in the Nordic region as well as post-learnings on planning and execution elements in order to have a successful joint procurement.

**Methodology:** The visualised model of a product life cycle (Figure 1) was applied to understand where a pilot of joint procurement would support the supply issues of older products. This led to a shared understanding between the countries on where supply issues may occur and potential solutions. An evaluation of establishing the joint procurement process in Amgros, which took approximately 2 years, is now available as a best practice with “Do’s and Don’ts” for other countries with interest in joint procurement. The criterion in the tenders was either price alone or in combination with qualitative criteria. One of the tenders included a mandatory bid for all 3 markets, the rest of the tenders were mandatory for Denmark and Norway with optional submission for Iceland. This was an outcome of hearings with suppliers. The feedback from the hearings was to modify the tender materials into a new proposal for suppliers. A political framework was signed between the countries to have a shared foundation to build on.

Region covered: Denmark (the joint procurement was performed with Norway and Iceland)

Time period: 2018-2019

**Results:** The final outcome of a joint procurement was evaluated. Evaluation of the submission and preparation part showed that the majority of joint tenders had an efficient competition on price with a representative amount of suppliers bidding. It also showed that it was vital to have collaboration and to listen to stakeholders in order to have a robust insight on what was possible for all involved parties. The thorough preparations supported the process and the final outcome. There was dual engagement between the stakeholders and transparency on the wish from countries to overcome barriers and conduct joint procurement to support supply issues.

**Conclusions and lessons learned:** Efficient and timely planning is crucial. Collaborations between the involved stakeholders are important. Mutual understanding of the interests and strategy is helpful in building a shared view on the problems and potential solutions. It is seen as essential, when planning joint procurement, to include logistic thinking already in the early tender planning phase.

**Funding Source**

Amgros I/S

**Keywords:** Joint procurement, best practice sharing, product life cycle, tendering

**Fig. 1 (abstract P25).**
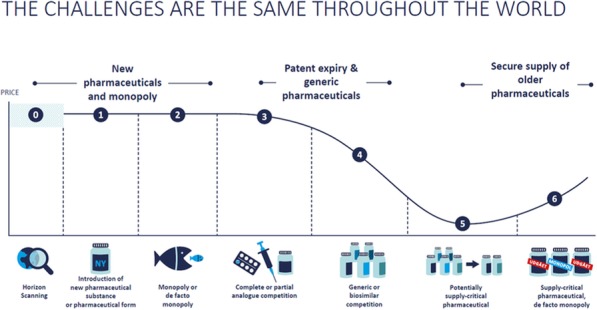
Product Life Cycle of Pharmaceuticals

